# Insights into synthesis and function of KsgA/Dim1-dependent rRNA modifications in archaea

**DOI:** 10.1093/nar/gkaa1268

**Published:** 2021-01-12

**Authors:** Robert Knüppel, Christian Trahan, Michael Kern, Alexander Wagner, Felix Grünberger, Winfried Hausner, Tessa E F Quax, Sonja-Verena Albers, Marlene Oeffinger, Sébastien Ferreira-Cerca

**Affiliations:** Regensburg Center for Biochemistry, Biochemistry III – Institute for Biochemistry, Genetics and Microbiology, University of Regensburg, Universitätsstraße 31, 93053 Regensburg, Germany; Institut de Recherches Cliniques de Montréal, 110 Avenue des Pins Ouest, Montréal, Québec H2W 1R7, Canada; Faculty of Medicine, Division of Experimental Medicine, McGill University, Montréal, Québec H3A 1A3, Canada; Département de Biochimie, Faculté de Médecine, Université de Montréal, Montréal, Québec H3T 1J4, Canada; Regensburg Center for Biochemistry, Biochemistry III – Institute for Biochemistry, Genetics and Microbiology, University of Regensburg, Universitätsstraße 31, 93053 Regensburg, Germany; Molecular Biology of Archaea, Institute of Biology II, Faculty of Biology, Microbiology, University of Freiburg, Freiburg, Germany; Chair of Microbiology – Institute for Biochemistry, Genetics and Microbiology, University of Regensburg, Universitätsstraße 31, 93053 Regensburg, Germany; Chair of Microbiology – Institute for Biochemistry, Genetics and Microbiology, University of Regensburg, Universitätsstraße 31, 93053 Regensburg, Germany; Archaeal Virus-Host Interactions, Institute of Biology II, Faculty of Biology, Microbiology, University of Freiburg, Freiburg, Germany; Molecular Biology of Archaea, Institute of Biology II, Faculty of Biology, Microbiology, University of Freiburg, Freiburg, Germany; Institut de Recherches Cliniques de Montréal, 110 Avenue des Pins Ouest, Montréal, Québec H2W 1R7, Canada; Faculty of Medicine, Division of Experimental Medicine, McGill University, Montréal, Québec H3A 1A3, Canada; Département de Biochimie, Faculté de Médecine, Université de Montréal, Montréal, Québec H3T 1J4, Canada; Regensburg Center for Biochemistry, Biochemistry III – Institute for Biochemistry, Genetics and Microbiology, University of Regensburg, Universitätsstraße 31, 93053 Regensburg, Germany

## Abstract

Ribosomes are intricate molecular machines ensuring proper protein synthesis in every cell. Ribosome biogenesis is a complex process which has been intensively analyzed in bacteria and eukaryotes. In contrast, our understanding of the *in vivo* archaeal ribosome biogenesis pathway remains less characterized. Here, we have analyzed the *in vivo* role of the almost universally conserved ribosomal RNA dimethyltransferase KsgA/Dim1 homolog in archaea. Our study reveals that KsgA/Dim1-dependent 16S rRNA dimethylation is dispensable for the cellular growth of phylogenetically distant archaea. However, proteomics and functional analyses suggest that archaeal KsgA/Dim1 and its rRNA modification activity (i) influence the expression of a subset of proteins and (ii) contribute to archaeal cellular fitness and adaptation. In addition, our study reveals an unexpected KsgA/Dim1-dependent variability of rRNA modifications within the archaeal phylum. Combining structure-based functional studies across evolutionary divergent organisms, we provide evidence on how rRNA structure sequence variability (re-)shapes the KsgA/Dim1-dependent rRNA modification status. Finally, our results suggest an uncoupling between the KsgA/Dim1-dependent rRNA modification completion and its release from the nascent small ribosomal subunit. Collectively, our study provides additional understandings into principles of molecular functional adaptation, and further evolutionary and mechanistic insights into an almost universally conserved step of ribosome synthesis.

## INTRODUCTION

The directed multi-step and sequence-specific peptide bond formation allowing the synthesis of protein from mRNA is a universally conserved process mediated by the heart of the translation process: the ribosome. Despite its central role in gene expression, individual components of the translation machinery and part of the translation mechanism itself differ between the different domains of life (1–4). The ribosome′s structural components [i.e. ribosomal RNAs (rRNA) and ribosomal proteins (r-proteins)] and its associated translation factors have evolved around a universally conserved structural core, to which either existing parts have been extended or deleted and new components have been added (1–4). This structural and compositional diversity has enabled the emergence of additional layers of function and regulation in the course of its evolution (1–4). Moreover, this diversity may have shaped how ribosomes are synthesized across the tree of life.

Strikingly, ribosome biogenesis in bacteria and eukaryotes differs profoundly, since only rare *trans*-acting factors were shown to be common to both the bacterial and eukaryotic ribosome biogenesis pathways (5–7), hence, suggesting that the ribosome synthesis process has been almost completely re-engineered between bacteria and eukaryotes (6,8). Yet, sequence prediction analyses of various archaeal genomes indicated that a few genuine eukaryotic and bacterial ‘specific’ ribosome biogenesis factor homologs are present in archaea [see (5,6), and Ferreira-Cerca laboratory, own unpublished observations]. These observations hinted that ribosome biogenesis in archaea may proceed with the help of eukaryotic- and bacterial-like features to which archaeal idiosyncrasies have presumably been added (6). In agreement with this notion, recent analyses, including those of selected putative ribosome biogenesis factor homologues and *cis*-acting elements, support this general framework (9–14). We have recently provided *in vivo* and *in vitro* insights into the function and regulation of the archaeal homologs of the Rio proteins (11), and importantly, this and other studies indicate a striking similarity between eukaryotic and archaeal late small ribosomal subunit (SSU) synthesis steps (5,6,9,11–14). Moreover, using a *cis*-acting element mutation approach, we have recently revealed the functional importance of an archaeal-specific step (10), thereby reinforcing the idea that ribosome biogenesis in archaea proceeds via a molecular patchwork, that comprises eukaryotic-like, bacterial-like and archaeal-specific steps (6). Despite these efforts, the *in vivo* archaeal ribosome biogenesis pathway remains essentially to be fully determined (6,15).

In this study, we report the functional analysis of the archaeal small ribosomal subunit rRNA dimethyl transferase KsgA/Dim1 (bacterial and eukaryotes name, respectively, also named RsmA in bacteria), an almost universally conserved ribosome biogenesis factor (16–19). KsgA/Dim1 performs dimethylation of two universally conserved adenosine residues located in the terminal 16S/18S rRNA helix (h45) (16,17,20,21). Although the rRNA modifications are non-essential for the viability of the bacterial or eukaryotic organisms analyzed to date, they are believed to fine-tune translation activity, thereby promoting translation accuracy and cellular fitness (16,22–26). Such purpose has been recently illustrated on the structural level, whereby bacterial KsgA-dependent modifications contribute to a rRNA modifications network which may stabilize the SSU A- and P-site architecture (27,28). Moreover, early studies demonstrated that absence of bacterial KsgA or its catalytic activity confers resistance to the aminoglycoside antibiotic kasugamycin (20,21,29). Lastly, beyond its established dimethyl transferase function, KsgA/Dim1 is also required for proper ribosome biogenesis, both in bacteria and eukaryotes (16,23,25,30). In bacteria, KsgA promotes late folding of the two last 16S rRNA helices, h44 and h45, and may contribute to the final steps of SSU biogenesis (27,31,32). In the yeast *Saccharomyces cerevisiae*, Dim1 is required as a structural component for early rRNA maturation and remains associated to late cytoplasmic pre-40S particles where it modifies the two previously mentioned conserved adenosines, and, in addition, is presumed to promote proper folding of h44/h45 (23,33,34). Release of Dim1, in this cellular context, is stimulated by the action of the adenylate kinase/ATPase, Fap7, in an ATP-dependent manner (33). In contrast, in human cells the dwell-time of KsgA/Dim1 on pre-ribosomal subunit differs from yeast, as several reports provided evidence for its release at an earlier stage of nascent pre-40S (35–37). The contribution of Fap7 to KsgA/Dim1 release in human cells has not been investigated to date. Taken together, results obtained from *Eschericha coli* and *S. cerevisiae* suggest a model where KsgA/Dim1-dependent dimethylations may at least to some extent, act as a late quality control checkpoint during SSU biogenesis (23,32,33,38). Whether this notion of a quality control checkpoint also exists in human cells awaits to be clarified.

In contrast to bacteria and eukaryotes, the role of archaeal KsgA/Dim1 for ribosome synthesis and function has not been clearly analyzed *in vivo*. Previous studies demonstrated that an archaeal KsgA/Dim1 homolog can functionally substitute the KsgA/Dim1 loss of function in bacteria, but not in eukaryotes (19,39). In fact, eukaryotic KsgA/Dim1 functional integrity requires the presence of additional eukaryote-specific elements in its N-terminus (39). More generally, the occurrence of the dimethylations across the tree of life has been assumed based on the presence of KsgA/Dim1 homologues, and less commonly on direct analysis of rRNA modifications (18,40–45). Yet, early pioneering studies from Carl Woese and collaborators indicated the presence of 16S rRNA dimethylations incongruities in some Methanogens (46). In addition, previous analysis of the rRNA modification-status of some organelles′ ribosomes suggested that KsgA/Dim1-dependent modifications can be absent or heterogeneous (47,48). Finally, recent analysis of the archaeal obligatory symbiont, *Nanoarchaeum equitans*, revealed an absence of KsgA/Dim1-dependent dimethylation of 16S rRNA in this organism (19). Collectively, these observations open the possibility that the amount and distribution of the KsgA/Dim1-dependent modifications in the different domains of life could be more diverse than previously thought. Furthermore, the functional implications of these incongruities on ribosome biogenesis and function have not been determined yet.

In this work, we performed functional analyses of archaeal KsgA/Dim1 to further unravel the archaeal ribosome biogenesis pathway. Our study reveals that archaeal KsgA/Dim1 is not essential for the viability of three representative evolutionary divergent archaea. However, the presence of archaeal KsgA/Dim1 provides a growth advantage under stress conditions, as illustrated by our detailed analysis in the model archaeon *Haloferax volcanii*. This observation correlates with organism-specific proteostasis deregulation in the absence of KsgA/Dim1, as shown by label-free quantitative proteomics analysis. Taken together, our results indicate that archaeal KsgA/Dim1 contributes to the efficient expression of a subset of the cellular proteome.

In addition, our study also provides a functional and phylogenetic-based analysis indicating that the efficiency of KsgA/Dim1-dependent h45 modifications varies in different organisms and that this disparity is intrinsically determined by inherent h45 sequence/structure and/or KsgA/Dim1 properties. In summary, our study provides a structural and functional account on how rRNA sequence/structural variability control the extent of the KsgA/Dim1-dependent modifications, and how it may contribute to shaping the KsgA/Dim1 functional adaptation and its association/dissociation landscape in the different domains of life.

## MATERIALS AND METHODS

### Strains, plasmids and growth conditions

Strains and plasmids used in this study are listed in Supplementary Tables S1 and S2, respectively.

*Haloferax volcanii* strains were grown in rich (Hv-YPC) or enhanced casamino acids (Hv-Ca^+^), or casamino acids (Hv-Ca) or minimal (Hv-min) media (49) at 42°C under vigorous agitation. For Tryptophan-based induction, genes under the control of the Tryptophanase promotor (p.*tna*), cells were cultivated in Hv-Ca and/or Hv-Ca^+^ in presence of minimal amounts of Tryptophan (50 μg/ml; ∼0.25 mM). Expression was induced by addition of indicated amounts of tryptophan.

*Sulfolobus acidocaldarius* [MW001 Δ*pyrE* – (50)] cells were grown in standard Brock medium (50,51) at 65°C under vigorous agitation.

*Halobacterium salinarum* (52) and *Halorubrum lacusprofundi* (53) cells were kindly provided by Prof. Dr Marchfelder (University of Ulm). *H. salinarum* and *H. lacusprofundi* were cultivated in modified Hv-YPC containing 27% salt water (w/v), as previously described (52–54).

*Pyrococcus furiosus* (55), *Archaeoglobus fulgidus* (56), *Methanothermobacter thermoautotrophicus* (57) and *Methanopyrus kandleri* (58) biomass and/or total RNA were generously provided by the Archaea Centre Regensburg.

*Bacillus subtilis* strain 168 (59) was kindly provided by Dr Patrick Babinger (University of Regensburg). Molecular cloning and amplification of plasmids were performed according to standard molecular biology methods.

### Construction of Δ*ksgA/dim1* in *H. volcanii*

Marker-less in-frame deletion was performed using the pop-in/pop-out strategy (60). In brief, 500 bp of the upstream (us) and downstream (ds) regions spanning the target open reading frame (*Hv*_KsgA = *HVO_2746*) were amplified by PCR. The resulting PCR product were cloned into the integrative vector pTA131 (49). The resulting integrative plasmid pTA131-*KsgA-usds* was transformed in H26 cells using the spheroplast/PEG transformation protocol (49). Positive transformants were first selected on Hv-Ca^+^ plates lacking uracil (pop-in). Recombination events generating Δ*ksgA/dim1* were selected on Hv-Ca^+^ containing 5-FOA (pop-out). Knock-out candidates (correct deletion and absence of the targeted ORF) were verified by PCR and Southern blot analysis.

To generate the reintegration strains, Δ*ksgA*::KsgA and Δ*ksgA*::*ksgA* E84A, plasmid pTA131-us_KsgA_ds and pTA131-us_*ksgA-E84A_*ds were generated and transformed into Δ*ksgA/dim1* cells. Recombination events generating Δ*ksgA*::KsgA and Δ*ksgA*::*ksgA* were selected on Hv-Ca^+^ containing 5-FOA (pop-out). Knock-in candidates were verified by PCR.

### Construction of Δ*ksgA/dim1* in *S. acidocaldarius*

Marker-less in-frame deletion was performed using the pop-in/pop-out strategy (50). In brief 500 bp of the upstream (us) and downstream (ds) regions spanning the target open reading frame (*Saci*_KsgA/Dim1 = *Saci_0623*) were amplified by PCR and cloned into the integrative vector pSVA431 (50). The resulting integrative plasmids pSVA431-*KsgA/Dim1 usds* was transformed in MW001 cells by electroporation as previously described (50). Positive transformants were first selected on Brock medium plates lacking uracil (pop-in). Recombination events generating Δ*ksgA/dim1* were selected on Brock medium plates containing 5-FOA (pop-out). Knock-out candidates (correct deletion and absence of the targeted ORF) were verified by PCR and Southern blot analysis.

### Construction of Δ*ksgA/dim1* in *P. furiosus*

Marker-less in-frame deletion was performed using the pop-in/pop-out strategy as previously described (61). To avoid conflicts with homologous recombination during the disruption procedure we also deleted Pf1864 encoding for one of multiple duplicated transposases present in *Pyrococcus furiosus* genome. Flanking upstream (us: ∼600 nt) and downstream (ds: ∼1000 nt) regions spanning the target open reading frame (*Pfu*_KsgA/Dim1 = *Pf1863 and Pf1864*) were amplified and assembled together with the counter-selection cassette (cs) by fusion PCR (61). The resulting PCR fusion was cloned into Zero Blunt™ TOPO™-Vector (Invitrogen) and transformed into the recipient strain MUR37Pf (61). Positive transformants were first selected on $\frac{1}{2}$ SME medium with 40 mM pyruvate, 0.1% yeast extract and 0.1% peptone, but in the absence of agmatine sulfate, inosine, and guanine (pop-in). Recombination events generating Pf1863(KsgA/Dim1)/Pf1864 deletion were selected on GELRITE-solidified plates containing $\frac{1}{2}$ SME medium with 40 mM pyruvate, 0.025% yeast extract, 8 mM agmatine sulfate and 50 μM 6-methylpurine for counter selection (pop-out). Knock-out candidates (correct deletion and absence of the targeted ORF) were verified by PCR analysis.

### Growth analysis of *H. volcanii, S. acidocaldarius and P. furiosus*

Semi-automated growth analysis of *H. volcanii* was performed as previously described (62). In brief, exponentially growing cells (Hv-YPC) were diluted in Hv-YPC and aliquoted into 96-well plates. Growth (OD_612nm_) at 41.5°C (±0.3°C) was monitored every 20 min for at least 2 days, using a TECAN Infinite F500 reader. Optical density values were corrected with the average background optical density measurement of abiotic medium. Growth analysis for each strain were performed in triplicate.

Growth analyses of *S. acidocaldarius* were performed manually as follows: exponentially growing cells (Brock medium supplemented with uracil) (50,51) were diluted in pre-warmed Brock medium and incubated at 65°C with agitation. Optical density (OD_600nm_) was measured at regular time interval.

Growth analyses of *P. furiosus* were performed as recently described (61). In brief, *P. furiosus* strains MURPf37 (parental strain) and MURPf81 (*Pf1863/64* deletion) were cultivated under anaerobic conditions in 40 ml $\frac{1}{2}$ SME medium supplemented with 8 mM agmatine sulfate, 8 mM inosine, 8 mM guanine, 0.1% yeast extract, 0.1% peptone and 40 mM pyruvate at 85°C to ensure heat stability of the different chemicals used over the time of the growth experiments (61). Growth was recorded in biological triplicates during 60 h of incubation by measuring the turbidity changes *in situ* using a photodiode and a LED with 850 nm as light source. The recorded values were converted to cell/ml by using a calibration curve with known cell concentrations, calculated in a Thoma counting chamber (0.02-mm depth; Marienfeld, Lauda-Königshofen, Germany) using phase-contrast microscopy (61).

### Competition assay

**Figure 1.**
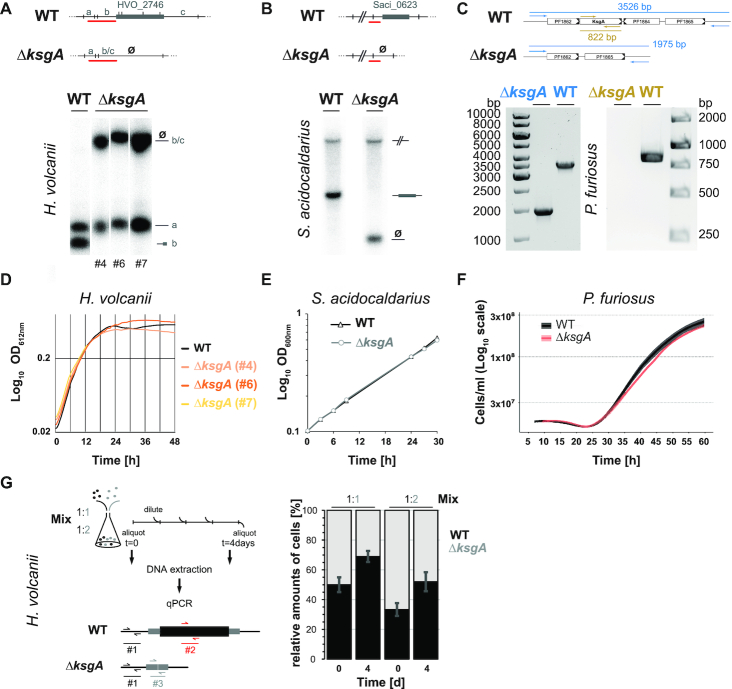
Archaeal KsgA/Dim1 is dispensable for cell viability. (**A**, **B**) Verification of KsgA/Dim1 in-frame deletion in *H. volcanii* and *S. acidocaldarius* by Southern blot analysis. Total genomic DNA from KsgA/Dim1 (HVO_2746 and Saci_0623) deletion candidates was extracted and digested with restriction enzyme at the specified positions (SalI for *H. volcanii* and PstI for *S. acidocaldarius* are indicated by perpendicular bars). The resulting DNA fragments were separated by agarose gel electrophoresis and immobilized on a membrane prior to hybridization with the indicated radioactively labeled probe (specified in red). The respective expected genomic organization, restriction sites, and radioactively labeled probe used are schematically depicted (upper panel). Phosphor-imaging analysis (lower panel) for (A) *H. volcanii* and (B) *S. acidocaldarius* deletion candidates are shown. #nr refers to different independent deletion mutants. (**C**) Verification of KsgA/Dim1 in-frame deletion in *P. furiosus* by diagnostic PCR analysis. Total genomic DNA of parental strain (MURPf37) and KsgA/Dim1 (PF1863) deletion candidate (MURPf81) was extracted and subjected to diagnostic PCR using the indicated primers. Expected length of the corresponding PCR products using the specified primers before and after deletion of PF1863/PF1864 are indicated (upper panel). The corresponding PCR reactions targeting the upstream PF1862 and downstream PF1865 regions (indicated in blue) or the KsgA/Dim1 ORF (PF1863) (in gold) were separated by agarose gel electrophoresis (lower left and right panel, respectively). (**D**) Growth analysis of cells lacking KsgA/Dim1 in *H. volcanii*. Exemplary growth analyses for wildtype parental strain (H26) and three independent KsgA/Dim1 deletion strains (clone #4, #6 and #7) are depicted. (**E**) Growth analysis of cells lacking KsgA/Dim1 in *S. acidocaldarius*. Exemplary growth analyses for wildtype parental strain (MW001) and KsgA/Dim1 deletion strain is shown. (**F**) Growth analysis of cells lacking KsgA/Dim1 in *P. furiosus*. Exemplary growth analysis for wildtype parental strain (MURPf37) and KsgA/Dim1 deletion strain (MURPf37) is provided. (**G**) Pairwise growth competition assays of *H. volcanii* wildtype and KsgA/Dim1 deletion strains. At the indicated time points, relative cell numbers were determined by quantitative PCR analysis using the portrayed strategy (see Materials and Methods for more details).

Logarithmically growing wildtype (H26) and KsgA deleted cells were diluted in fresh YPC medium and mixed in 1^WT^:1^Δ^*^ksgA^* or 1^WT^:2^Δ^*^ksgA^* (based on OD_600 nm_). The resulting cell mixtures were diluted in fresh YPC medium every day. Cells were collected at the indicated time points and their relative amount was determined by quantitative PCR analysis. Genomic DNA was extracted by DNA spooling (https://haloarchaea.com/halohandbook/) (54) and serial dilution were subjected to qPCR analysis using the indicated primers (Supplementary Table S3). Reactions were performed in a total volume of 20 μl using Sybr Green I (Roche) and Taq Master Mix kit (Qiagen) as suggested by the manufacturer, except that 2.5 mM MgCl_2_ were used. After a 5 min initial denaturation at 95°C, 35 cycles of 10 s at 95, 55 and 72°C each was performed and fluorescence measurement were recorded at the end of the elongation step on a Rotor-Gene Q system (Qiagen). Specificity of amplifications were ensured by melting curve analysis of the individual samples after completion of the PCR amplification. Short elongation times were chosen to selectively amplify shorter amplicons. The cycle threshold (C_T_) values of individual amplification were determined with the Rotor-Gene 6000 software (Qiagen). Quantifications were performed as described earlier (62,63). Total cell population was defined as the C_T_ value obtains for an arbitrary DNA region located in closed proximity of the KsgA ORF (*HVO_2746)* (amplicon #1 in Figure 1G, amplicon size: 134 bp) which is common to both WT and Δ*ksgA*. Relative amounts of KsgA deleted cells was defined by the C_T_ value obtained for a deletion strain-specific short amplicon surrounding the deleted KsgA ORF (amplicon #2 in Figure 1G, amplicon size: 126 bp). Relative amount of Wildtype cells was defined by a short amplicon amplifying the KsgA ORF (amplicon #3 in Figure 1G, amplicon size: 146 bp) and additionally verified as the relative differences of the C_T_ values obtained for KsgA deleted strain population and the total amount of cells. Relative amounts were normalized to *t* = 0. Experiments were performed in biological duplicates. Serial dilutions (*n* = 2) of extracted DNA were analyzed in technical duplicate.

### Motility analysis

For motility analysis, semi-solid motility plates containing the indicated media and a reduced agar concentration (0.3%) were used as described previously (64). Exponentially growing cultures were adjusted to similar cell density (OD_600nm_ = 0.3), and 4 μl were spotted onto the agar surface. Cell migration was evaluated in ‘Fiji’. The mean value of the biological and technical replicates is provided. The average values for each biological replicate were compared using an unpaired *t*-test. The respecting averages are plotted on top of the individual values and visualized as described previously (65).

### Adhesion assay

Adhesion assay was adapted from (66,67). Exponentially growing cells in Hv-YPC, Ca and Ca^+^ -media were adjusted to equal OD_600 nm_ of 0.3 and then distributed as 150 μl aliquots (at least three technical replicates) in a standard 96-well plate with a lid. The outer rows were not inoculated with cells and were used as media background blanks. The well plate was then incubated under vigorous agitation for ∼8 h at 42°C. Adhesion was stimulated by incubating cells for 40 h at 42°C without agitation. Non-adherent cells were removed, and the remaining adherent cells were fixed with 200 μl of 2% acetic acid in 20% salt water for 4 min at room temperature. The adhering cells were stained with 200 μl of a 0.2% crystal violet solution for 10 min. Three steps of rinsing with water preceded air drying for at least 1 h up to over-night. Once fully dried, the stained adherent cells were resuspended with 200 μl of a 10% acetic acid and 30% MeOH solution and shake for 5 min before measuring in a TECAN reader at OD_612 nm_. Individual values were tested for significance using an unpaired *t*-test. Results were plotted and visualized as described previously (65).

### Cell morphology analysis

*H. volcanii* cultures were grown in Hv-Ca media for two serial dilutions over two days and imaged at low (OD < 0.04) and higher (OD > 0.04) densities on 1% agarose–18% salt–water pads. The cells were grown and observed on two independent occasions. Image acquisition was performed on a Zeiss Microscope at a magnification of 100× using PH3 setting. Cell morphology analysis was done with the ‘MicrobeJ’ plugin for Fiji (68,69) and was manually curated for outliers or false detections. The parameters for MicrobeJ were the following: 100× Magnification Scale, 0.065 pixel/μm (65). Significance between the morphology data for merged replicates of WT cells compared to Δ*ksgA* cells under low and high densities was determined by Wilcoxon rank-sum test with continuity correction.

## BONCAT

Detail implementation of Bioorthogonal Non-canonical Amino Acid Tagging (BONCAT) of *H. volcanii* is described in (70) and is essentially adapted from (71,72). In brief, *H. volcanii* cells were grown in Hv-min and pulse labeled with the methionine surrogate, L-AHA, for the indicated time points. After protein extraction, reduction and alkylation steps, the L-AHA proteins were labeled with Cy5.5 or Cy7 using strain-promoted azide-alkyne click-chemistry (SPAAC) using DBCO-Cy5.5 or DBCO-Cy7. Protein were separated by 1D or 2D SDS-PAGE and fluorescent signals were visualized using an Odyssey-LICOR instrument. For 2D-SDS PAGE, first dimension isoelectric focussing was performed with a pH 3–6 strip (BioRad) according to the manufacturer′s recommendations.

### Label-free proteomics and bioinformatic analyses

Cell pellets were suspended in 500 μl of Extraction Buffer (EB: 150 mM NaCl, 100 mM EDTA, 50 mM Tris pH 8.5, 1 mM MgCl_2_) supplemented with 1% SDS and incubated 13 min at 95°C. After 5 min of cooling at RT, cell lysates were clarified for 10 min at 16 000 g. Solubilized proteins were purified by methanol/chloroform extraction. Purified proteins were reduced in the dark for 1 h in EB supplemented with 2% β-mercaptoethanol. Reduced proteins were precipitated by addition of 4 volume acetone at −20°C for at least 2 h and centrifuged at 4°C with 16 000 g for 10 min. Supernatants were removed and pellets were washed twice with −20°C acetone and centrifuged as before (73).

Pellet were dried and processed as following. Dried pellets were resuspended in 80 μl of Tris 100 mM pH 8.0 containing 0.2% ProteaseMax (Promega) and incubated in a ThermoMixer for 60 min at 30°C with agitation set at 1400 rpm. Samples were cleared by centrifugation at 16 000 g for a minute, then diluted with Tris 100 mM pH 8.0 to reach a ProteaseMax final concentration of 0.04%. Proteins were measured by Bradford, and 20 μg of each sample were reduced with 5 mM DTT for 30 min at 37°C, followed by cysteines alkylation with 15 mM Iodoacetamide in the dark for 30 min prior to trypsin digest at a ratio of 1:20.

Due to severe ion suppressing signals observed in the KsgA WT and mutant's reintegration samples while using a C18 SPE for samples cleanup, all samples were instead cleaned on a 2 mg sorbent 96-well plate Oasis MCX μElution plate (Waters). Briefly, the samples were loaded, and successively washed with 500 μl 0.1% TFA, 500 μl 80% ACN, 0.1% TFA and 500 μl water. Samples were eluted with 10% NH_4_OH in 90% methanol and brought to dryness. Peptides were resolubilized in 2% ACN and 1% FA. Samples were loaded at 400 nl min^−1^ on a 17 cm × 75 μm i.d. PicoFrit fused silica capillary column (New Objective), packed in-house with Jupiter 5 μm C18 300 Å (Phenomenex). The column was mounted in an Easy-nLC II system (Proxeon Biosystems) and coupled to an Orbitrap Fusion mass spectrometer (ThermoFisher Scientific) equipped with a Nanospray Flex Ion source (Proxeon Biosystems). Peptides were eluted at a flow rate of 200 nl min^−1^ on 2-slope gradient made with 0.2% FA in water (buffer A) and 0.2% FA in 100% ACN (buffer B). Concentration of buffer B first increased from 2% to 36% over 165 min and from 36% to 90% over 15 min.

The mass spectrometer was operated in data-dependent acquisition mode. Full MS scan in the range of 360–1560 *m*/*z* range were acquired in the Orbitrap at a resolution of 120 K. AGC target was set to 2.5e5 with a maximum fill time of 50 ms. A top speed cycle mode was used, in which +2 to +5 ions were isolated in the Quadrupole using a window of 1.6 Da and fragmented in the HCD collision cell (collision energy set at 29%) before being detected in the linear trap. The MS2 AGC target was set to 1.5e4 with a maximum fill time of 50 ms. The lock mass option (lock mass: *m*/*z* 371.101233) was used for internal calibration.

Label-free quantification was done with MaxQuant v1.6.14 using the default LFQ settings (74,75). The Trypsin/P cleavage rule was applied, allowing two miscleavages for the analysis. Methionine oxidation and protein N-term acetylation were set as variable modification while carbamidomethylation of cysteines was set as fixed modification. Raw results from archaeal material were searched against a recently described Fasta database (73) (https://github.com/arcpp/ArcPP/tree/master/databases), while bacterial material was searched against the *E. coli* K-12 strain reference Fasta database (UP666666625_83333.fasta). A minimum of seven amino acid peptides were considered up to 6250 Da. The default FDR for PSM, protein and decoy were left unchanged at 1%, and a minimum of two unique and razor peptides were used for quantification. Perseus v1.6.12 was used to remove contaminants, decoys and peptides only identified by site. The remaining values were log_2_ transformed, and missing values (null values) were implemented separately for each column using the Perseus processing engine (option replace missing values from normal distribution). Note that in the case of KsgA/Dim1 knock-out (null values), all imputed values are arbitrary and provide a largely underestimated view of the KsgA/Dim1 fold change, and is only provided to facilitate general analysis of the full dataset. A two-tailed *t*-test was applied to the data, and proteins showing relative expression changes with *P-*values <0.05 were retained for further analysis. Results are summarized in Supplementary Tables S4–S6.

ArCOG annotations (76,77) (ftp://ftp.ncbi.nih.gov/pub/wolf/COGs/arCOG/ar14.arCOGdef.tab; 10-08-2019 version) were manually added to the MaxQuant output file while other annotations were added through Perseus (78) (http://annotation.perseus-framework.org).

The mass spectrometry proteomics data have been deposited to the ProteomeXchange Consortium (http://www.proteomexchange.org/) via the PRIDE partner repository (79) with the dataset identifier PXD021827 and doi:10.6019/PXD021827.

Enrichment analysis of archaeal and bacterial cluster of orthologous genes (arCOGs, COGs) was performed by i) extracting the gene specific arCOG/COG information from the respective database (ftp://ftp.ncbi.nih.gov/pub/wolf/COGs/arCOG/ and ftp://ftp.ncbi.nih.gov/pub/COG/COG2020/data) and ii) performing gene set enrichment using the goseq package in R, which calculates enrichment probabilities after correcting for gene lengths and multiple testing (76,77,80,81).

Start codon analysis was performed by comparing the relative number of open reading frame starting with an AUG to the number of open reading frame starting with a non-AUG.

### RNA extraction

Unless stated-otherwise, total RNA was extracted using the hot-phenol extraction procedure as previously described (62,82).

### Primer extension analysis

For primer extension around 1–2 μg DNAse-treated total RNA were mixed with the appropriate labeled primers (1 pmol) (Supplementary Table S3), 40 mM dNTPs in a total volume of 6.5 μl and denatured for 5 min at 65°C. For read through-analysis with ddCTP, same conditions were used except that, dCTP was omitted and replaced by ddCTP. After 5 min, samples were put on ice and 2 μl 5× first-strand buffer, 0.5 μl 0.1 M DTT, 0.5 μl RNAsin and 0.5 μl SuperScript™ III RT (200 units/μl) was added. Samples were incubated for 1 h at 50–55°C depending on the primer used. Reaction were terminated by incubation at 70°C for 10 min. After the final denaturation step, RNA hydrolysis with 1 μl NaOH (1 M) and 0.25 μl EDTA pH 8.5 (0.5 M) was performed at 60°C for 30 min. The reaction was then neutralized with 1 μl HCl (1 M). An equal volume of primer extension loading buffer was added to the finalized reaction.

Sequencing ladder were generated with Thermo Sequenase Cycle Sequencing Kit with a slightly modified protocol. Sequencing Master mix containing 1 μg template DNA, 1 pmol of labeled-primer, 2 μl Reaction Buffer, and 2 μl of Thermo Sequenase DNA polymerase was completed to 17.5 μl with H_2_O. 4 μl of the Master Mix was added to 4 μl of each ddNTP mix (300 μM each of dATP, dCTP, dTTP and 7-deaza-dGTP, and 3 μM of the corresponding ddNTP). Single strand synthesis was run on a PCR-Cycler with the following settings: 3 min initial denaturation at 94°C, followed by 55 cycles of (30 s at 94°C, 30 s at 55°C and 60 s at 72°C). The finished reaction was then complemented with 4 μl of primer extension loading buffer.

Around, 5–10% of the primer extension reaction and around 10% of the sequencing ladder reactions were loaded onto either a small 14% Polyacrylamide 1× TBE–6 M urea gel (Novex Gel cassette, 1 mm thickness) or a medium sized very thin gel (20 cm × 17 cm, thickness ≤ 0.1 mm). The smaller gels were run at 200 V until the bromophenol blue almost exited the gel. The large gels were pre-run for 15 min at a constant 20 W and the actual run was performed at 25 W for around 45 min.

### Determination of relative mRNA levels by quantitative real-time PCR

Quantitative PCR analysis were essentially performed as described previously (83). RNA was extracted from 4 ml logarithmically growing cells in Hv-YPC medium (OD_600nm_ = 0.5) using RNeasy Mini-Kit (Quiagen #74106) as recommended by the manufacturer instructions. Three microgram of DNAse treated RNA was used for cDNA synthesis (10 min at 45°C) using QuantiNova™ Reverse Transcription Kit (Quiagen #205413) according to the manufacturer′s protocol. Quantitative real-time PCR was performed on 50 ng of cDNA using the qPCRBIO SyGreen Mix Lo‐ROX Kit (PCRBIOSYSTES) in a final volume of 10 μl in a Magnetic Induction Cycler (MIC) (Bio Molecular Systems) using the following settings: 2 min initial denaturation at 95°C following 60 cycles of 95°C 5 s and elongation for 30 s at 65°C. PCR amplification reactions targeting two regions of ArlA1 mRNA (*HVO_1210*, amplicon size: 139 and 126 bp), and one of ArlA2 *(HVO_1211*, amplicon size: 142 bp) and Gyrase B (*HVO_1572*, amplicon size: 140 bp) subunit were performed using the primers indicated in Supplementary Table S3. Specificity of the individual amplified product was validated by melting curve analysis. Analysis was done in technical quadruplicates. The cycle threshold (C_T_) values of individual amplification were determined by the accompanying dedicated software micPCR version 2.4.0 (Bio Molecular Systems).

### Expression and purification of recombinant proteins

Expression and purification of recombinant proteins were essentially performed as described previously (11,84,85)

In brief for purification of *Hv*_KsgA/Dim1 and *Hv*_KsgA/Dim1 E84A, induction was performed over-night at 20°C in presence of 0.1 mM IPTG. Cells were resuspended in K1800 high-salt buffer (20 mM Tris–HCl pH 7.5, 1.8 M KCl, 50 mM MgCl_2_, 10 mM imidazole, 10% glycerol) and lysed with zirconia beads.

After zirconia beads extraction, lysates were shortly clarified (5 min at 1000 rpm) prior to a second clarification step (10 000 rpm for 20 min).

Soluble His-tagged proteins were then immobilized on Talon-beads (Clontech) and extensively washed (20 mM Tris–HCl pH 7.5, 1.8 M KCl, 50 mM MgCl_2_, 10 mM imidazole). The proteins were eluted using Imidazole (elution buffer: 20 mM Tris–HCl pH 7.5, 1.8 M KCl, 50 mM MgCl_2_, 150 mM imidazole). Eluted proteins were buffer-exchanged [storage buffer: 20 mM Tris–HCl pH 7.5, 1.8 M KCl, 50 mM MgCl_2_, 10% glycerol (w/v)] and concentrated on ultrafiltration column to reduce the amounts of imidazole, aliquoted and stored at −80°C

### Reconstitution of KsgA/Dim1-dependent dimethylation *in vitro*

Cell pellets were resuspended in K1800-Buffer (20 mM Tris–HCl pH 7.5, 1.8 M KCl, 50 mM MgCl_2_,10% glycerol) and disrupted with an equivalent volume of zirconia beads using a Precellys device (Bertin Instruments) [3 times 5 cycles (30 s 6000 rpm and 30 s pause) at 4°C). The obtained lysates were centrifuged for 10 min at 4000 g 4°C and the supernatant was then cleared with a second centrifugation step (15 000 g for 30 min). Fifteen OD_260 nm_, as determined by Nanodrop measurement, were loaded onto a 5–30% (w/v solved in K1800-Buffer) sucrose gradient and centrifuged in SW40 either at 39 000 rpm for 4 h at 4°C or 16 h at 26 000 rpm at 4°C. The fractions containing the 30S ribosomal subunits were collected and pooled. WCE and 30S fractions were supplemented with excess recombinant Hv_KsgA/Dim1 (WT or catalytic mutant E84A) with or without 1 mM *S*-adenosyl-methionine as following.

Nucleic acids content of the WCE was measured on a Nanodrop-1000 at 260 nm and the relative amounts of genomic DNA, and rRNAs was estimated by agarose gel electrophoresis [note that a large amount (up to 50%) of genomic DNA is typically obtained]. Around one-third of the estimated RNA was considered to be 16S rRNA (30S). For *in vitro* reconstitution, ∼50 μg of nucleic acids (*A*_260 nm_) of WCE per reaction were used, corresponding to an estimated amount of ∼8.3 μg (∼17 pmol) 16S rRNA. Purified recombinant protein concentration was determined by Nanodrop measurement with the following settings: Mol weight: ∼32 kDa, Ext coeff: 7575 M^−1^ cm^−1^. Around ∼1.5 nmol of purified recombinant protein was used (∼90-fold excess).

Similar conditions were used for the *in vitro* reconstitution with purified 30S fractions. Nucleic acid content from pooled sucrose gradients fractions were measured with a Nanodrop at OD_260 nm_. The amount of contaminating genomic DNA co-sedimenting in the 30S fraction was estimated by agarose gel electrophoresis (typically ∼50%). Around 30 μg of nucleic acids-equivalent of pooled 30S fractions were used for the *in vitro* reconstitution, corresponding to an estimated amount of 15 μg of 16S (30S) rRNA (∼31 pmol). Around ∼1.5 nmol of recombinant protein was used (∼45-fold excess).

RNAs were extracted and similar amounts of 16S rRNA were subjected to primer extension analysis as described above.

### KsgA/Dim1 release and co-sedimentation assays

Cell pellets were resuspended in K1800-buffer and disrupted with an equivalent volume of zirconia beads using a Precellys device (Bertin Instruments) [3 times 5 cycles (30 s 6000 rpm and 30 s pause) at 4°C]. The obtained lysates were centrifuged for 10 min at 4000 g 4°C and the supernatant was then cleared with a second centrifugation step (15 000 g for 30 min). For release assay 50 μg nucleic acids, as determined by Nanodrop measurement (see *in vitro* reconstitution above). Excess (∼100-fold molar excess) recombinant *Hv*_KsgA/Dim1 with or w/o 0.5 mM SAM were incubated at 42°C for 30 min. The reactions or whole cell extract (co-sedimentation assay) were loaded onto a 5–30% (w/v solved in K1800-buffer) sucrose gradient and centrifuged in SW40 either at 39 000 rpm for 4 h at 4°C or 16 h at 26 000 rpm at 4°C. Fraction collection (500 μl/fraction, speed: 1 ml/min) was performed on BioRad Biologic LP and data was recorded with LP-Dataview (BioRad) as previously described (86). 100 μl of each fraction were MeOH/CHCl_3_ precipitated and pooled together in soluble, 30S and 50S fractions according to the recorded UV trace. Protein were separated on an SDS-PAGE and KsgA/Dim1 was detected using HisProbe-HRP (Thermofischer Scientific) and chemiluminescence.

### Sequence alignments and 2D RNA structure prediction

A total of 1042 archaeal 16S rRNA sequences were obtained from the SILVA database (SSU r138.1; GTBD Taxonomy) (87) and aligned using Clustalw with default setting in the Jalview environment (88). The Sequences were manually curated and trimmed to only include h45. The consolidated sequence alignment, comprising a total of 742 remaining sequences, were grouped based on sequence homology. Representative sequences for each group were extracted and subjected to 2D RNA structure prediction using the ViennaRNA web services employing default parameters, and with the option set on no G•U closing at stem ends allowed (89). Phylogenetic classification of individual group members was curated manually and is summarized in Figure 6 and Supplementary Figure S3.

Likewise, 16S/18S rRNA sequences for bacterial (∼2700 sequences) and eukaryotic (∼20 000 sequences) organisms, respectively, were obtained from the SILVA database (SSU r138.1) (87). Note that to simplify the analysis, SSU rRNA sequences derived from chloroplasts and/or mitochondria were excluded. The obtained results are summarized in Supplementary Figure S4.

### Structural probing experiments

CMCT based chemical footprinting was performed on ‘native’ ribosomal particle and deproteinized total RNA. For native CMCT, chemical modifications were performed *ex vivo* as CMCT performed poorly *in vivo*. In brief, 30 OD_600 nm_ were centrifuged and resuspended in 500 μl K1800 buffer and lysed as described above. Nucleic acid concentration was determined by nanodrop measurement and concentration was adjusted 2–3 μg nucleic acids equivalent per μl lysate. Five microliters lysate were supplemented with 20 μl stabilization buffer (250 mM Boric acid dissolved in K1800 adjusted to pH 8.0) and 25 μl K1800 buffer and pre-incubated 20 min at 20°C. 2× concentrated CMCT working solution dissolved in K1800 buffer was freshly prepared. One volume of two times concentrated CMCT working solution dissolved in K1800 buffer was added to one volume of samples, and incubated for 20 min at 20°C.

For CMCT modification on extracted total RNA, 5 μl (∼3 μg/μl) of extracted RNA were mixed with equal volume of 4× CMCT Buffer (200 mM Borax, 20 mM MgCl_2_, 400 mM KCl and incubated for 20 min at 20°C. One volume of 2× concentrated CMCT working solution dissolved in water was added to the samples and incubated for 20 min at 20°C. The resulting modified RNA was directly purified as described above and analysed by primer extension analysis.

*In vivo* SHAPE analysis was performed as described recently (90).

### Ribosomal DNA variants co-expression and shuffling experiment in *E. coli*

Ribosomal DNA-shuffle strain AVS69009 [rDNA deletion strain complemented by pHK-rrnC (conferring Kanamycin resistance; Kan^R^)] and shuffle plasmid pHK-rrnC C1192U (conferring Ampicillin and Spectinomycin resistance: Amp^R^; Spc^R^, respectively) were described previously and kindly provided by Dr Vila-Sanjurjo (91–93). The indicated nucleotide exchanges were introduced in the shuffle plasmid pHK-*rrnC* C1192U. The resulting plasmids were transformed in the rDNA-shuffle strain. For co-expression analysis, Amp^R^/Kan^R^ cells were selected. For rDNA-shuffling experiments, Amp^R^ transformants were grown several times in LB-Amp containing medium and Amp^R^/Spc^R^ and Kan^S^ clones were selected.

## RESULTS

### Archaeal KsgA/Dim1 is not essential for cell viability

To shed light on the function of archaeal KsgA/Dim1, we attempted to generate knockouts in three different available genetic systems that encompass archaeal organisms of diverse lifestyle and evolution trajectories. We chose, the halophilic Euryarchaeon *H. volcanii* (94), the hyperthermophilic Euryarchaeon *P. furiosus* (55) and the thermoacidophilic Crenarchaeon *S. acidocaldarius* (51).

We first performed in frame marker-less gene-deletion of KsgA/Dim1 in *H. volcanii*, using the well-established pop-in/pop-out system (49,95). Several independent KsgA/Dim1 deletion mutants were obtained, as demonstrated by PCR and/or Southern blot analyses (Figure 1A), indicating that KsgA/Dim1 is not essential for *H. volcanii* cellular viability. To confirm that this feature is not limited to this organism, we deleted the KsgA/Dim1 open reading frame in the distantly related archaeon *S. acidocaldarius* (50). Likewise, we obtained independent clones that were lacking *Saci*_KsgA/Dim1 (Figure 1B). Lastly, we successfully generated a marker-less gene deletion in the hyperthermophile *P. furiosus* (Figure 1C). Subsequent growth analysis of the respective deletion strains showed similar growth behaviors between the respective isogenic wildtype and KsgA/Dim1 deleted strains in the laboratory growth conditions tested (Figure 1D-F). Together these results indicate that like its bacterial counterpart, archaeal KsgA/Dim1 is not essential for cell viability in laboratory conditions across evolutionary divergent organisms.

Since KsgA/Dim1 is well conserved we considered that despite the absence of significant differences on growth behavior (in optimal laboratory growth conditions) the presence of KsgA/Dim1 should provide a growth advantage in more selective conditions. To test this possibility, we performed pairwise competition analysis of wildtype and KsgA/Dim1 deleted strains using *H. volcanii* as a model system. As shown in Figure 1, KsgA/Dim1 provides a cellular fitness advantage in these competitive growth conditions (Figure 1G).

### *Haloferax volcanii* KsgA/Dim1 facilitates cell motility

**Figure 2.**
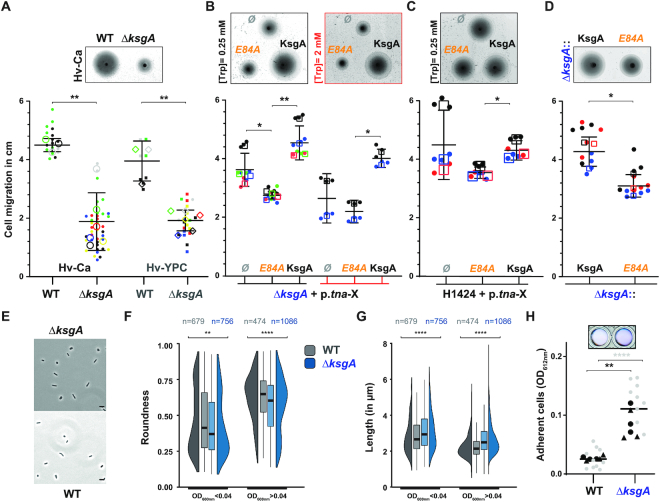
Archaeal KsgA/Dim1 facilitates cellular adaptation to environmental changes. (**A**) Influence of KsgA/Dim1 deletion on directional movement of *H. volcanii*. Motility assay of parental wildtype and KsgA/Dim1 deletion strains spotted on semi-solid agar plates containing Hv-Ca or Hv-YPC medium is depicted. A representative motility behavior of wildtype and KsgA/Dim1 deletion strain on Hv-Ca medium is provided (upper panel). Superplot analysis summarizing the motility behaviour of wildtype and KsgA/Dim1 deletion strains on Hv-Ca and Hv-YPC medium is shown (lower panel). All individual data points (dot) were plotted onto a scatter plot. Each colour corresponds to the results obtained for a biological replicate. The corresponding technical replicates indicated as individual dot of similar colour were averaged. The respective average is depicted as larger hollow with similar colour. The mean and standard deviation of the averaged values are indicated as black bars. Statistical significance was determined using an unpaired *t*-test of the biological averages (hollow shapes) (** *P* < 0.01). (**B**) Effect of KsgA/Dim1 complementation on directional movement of *H. volcanii*. KsgA/Dim1 deletion strains were transformed with the indicated pTA1228 plasmids derivative (p.*tna*-X) carrying KsgA or *ksgA*-E84A under the control of the tryptophan inducible tryptophanase promoter (p.*tna*). Ø denote strains transformed with an empty plasmid. Motility behaviour was analysed as in **A**). A representative motility behavior of cells expressing the indicated KsgA allele spotted on semi-solid agar medium containing the indicated concentrations of Tryptophan is provided (upper panel). (* *P* < 0.05 and ** *P* < 0.01). (**C**) Effect of KsgA/Dim1 overexpression on directional movement of *H. volcanii*. Strain H1424 (98) was transformed with the indicated pTA1228 plasmids derivative (p.*tna*-X) carrying KsgA or *ksgA*-E84A under the control of the tryptophan inducible tryptophanase promoter (p.*tna*). Motility behaviour was analysed as in (A). Ø denote strains transformed with an empty plasmid. A representative motility behavior of cells expressing the indicated KsgA allele spotted on semi-solid agar medium containing 0.25 mM Tryptophan is provided (upper panel). (* *P* < 0.05). (**D**) Influence of KsgA/Dim1 catalytic activity on directional movement of *H. volcanii*. KsgA/Dim1 wildtype (Δ*ksgA*::KsgA) or catalytic mutant (Δ*ksgA*::*ksgA* E84A) were genomically re-integrated in KsgA/Dim1 deletion strain. Motility behaviour was analysed as in (A). A representative motility behavior of reintegrated wildtype and KsgA/Dim1 catalytic mutant on Hv-Ca medium is provided (upper panel). (* *P* < 0.05). (E–G) Effect of KsgA/Dim1 on cellular morphology. (**E**) Exemplary field overview of cell morphology analyzed by phase contrast microscopy of parental strain and deleted for KsgA/Dim1 are depicted. Scale bar = 1 μm. (**F**) Comparison of WT (gray) and Δ*ksgA/dim1* (blue) cell roundness and (**G**) cell length determined at low (OD_600 nm_< 0.04) and high (OD_600 nm_> 0.04) cell densities. The morphology feature distributions for the strains at each condition are shown as half-violin and boxplots. Statistical significance (** *P* < 0.01 and **** *P* < 0.0001) was determined by Wilcoxon rank-sum test with continuity correction, with (n) corresponding to the total number of observations in the merged replicates. (**H**) Effect of KsgA/Dim1 on *H. volcanii* adhesion property. Adhesion assay of parental wildtype and KsgA/Dim1 deletion strains was performed as described in material and methods. Exemplary crystal violet stained cells adhering (upper panel) and superplot analysis summarizing the adhesion property of wildtype and KsgA/Dim1 deletion strains grown in Hv-Ca (lower panel) is shown. Relative amounts of adhered cells were determined at OD_612nm_ (see Materials and Methods). All individual data points (small points) were plotted onto a scatter plot as described in (A). Each colour corresponds to the results obtained for a biological replicate performed on the same day. (** *P* < 0.01 and **** *P* < 0.0001).

In their environment, prokaryotic cells sustain a balance between motile (swimming/swarming) and non-motile (biofilm formation/adhesion) growth enabling their fast adaptation to local environmental changes (96,97). To analyze the contribution of KsgA/Dim1 to general cellular adaptation, we first performed swarming assays on semi-solid agar plates containing different carbon/nitrogen sources. As shown in Figure 2, *H. volcanii* KsgA/Dim1 deleted strains were significantly impaired in their ability to swarm in comparison to the isogenic wildtype (H26) (Figure 2A). To further substantiate the KsgA/Dim1 involvement in swarming efficiency, we performed a complementation analysis of the deleted strain. For this purpose, we ectopically (over-) expressed KsgA/Dim1 wildtype and a putative catalytic mutant (E84A) under the control of the tryptophan-inducible tryptophanase promoter (98,99). Whereas expression of wildtype KsgA/Dim1 improved swarming efficiency of the mutant strain, expression of the KsgA/Dim1 catalytic mutant, in the deletion background, further reduced the swarming efficiency, compared to a deletion strain carrying an empty vector (Figure 2B). Moreover, reduced swarming efficiency was observed either after over-expression of KsgA/Dim1 catalytic mutant in a wildtype strain background (Figure 2C), or after genomic reintegration of the KsgA/Dim1 catalytic mutant (Figure 2D).

Recently, it has been observed that the motility efficiency of *H. volcanii* cells correlate with their apparent cellular morphology. Rod-shaped cells are generally motile, whereas rounded cells are less motile (64,100). Moreover, cell morphology/motility distribution varies at the population level depending on growth conditions and cell density (64,101). For example, in an early stage of cellular growth (OD_600 nm_ < 0.04) *H. volcanii* cells are more motile and thus predominantly appear rod-shaped, whereas at later time-point during the growth phase (OD_600_ _nm_ > 0.04) rounded, less-motile cells are accumulating (64). To analyze the effect of KsgA/Dim1 deletion on cell morphology in *H. volcanii*, we performed quantitative cell microscopy analysis at different time points of cellular growth and determined their respective cellular morphology distribution. In comparison to its isogenic wildtype, KsgA/Dim1 deleted strain exhibited an increased fraction of cells that were longer and less round (rod-shape) in early growth phase, and a delayed transition toward round cells formation in the later growth phase (Figure 2E-G). These results indicate that cellular morphology dynamic of *H. volcanii* cells is perturbed in the absence of KsgA/Dim1.

Finally, we analyzed the adhesion aptitude of *H. volcanii* KsgA/Dim1 deleted cells. Our results substantiate a moderate but reproducible increase in the capability of the deleted KsgA/Dim1 strain to adhere on the surface (Figure 2H).

Collectively, these results suggest that the absence of archaeal KsgA/Dim1 has a broad effect on cellular adaptation/physiology of *H. volcanii* cells.

### Archaeal KsgA/Dim1 sustains translation homeostasis of a subset of proteins

**Figure 3.**
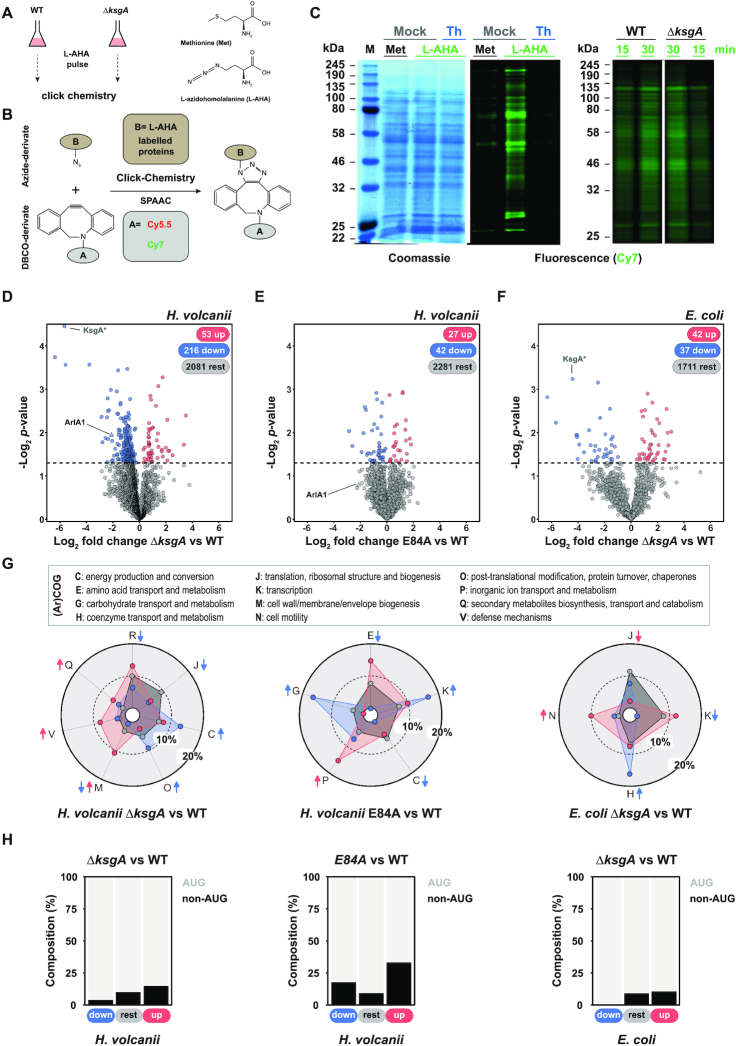
KsgA/Dim1 sustains translation homeostasis of a subset of proteins. (A, B) Principle of Bio-Orthogonal Non-Canonical Amino acid Tagging (BONCAT) analysis. (**A**) Cells are pulse-labeled with the methionine surrogate, l-azidohomoalanine (L-AHA). (**B**) The resulting azide-containing proteins can be labeled by click-chemistry (Strain-Promoted Alkyne-Azide Cycloadditions: SPAAC) with DBCO-derivative, like cyanine fluorophores. (**C**). Effect of translation inhibitor and KsgA/Dim1 deletion on bulk protein synthesis. Incorporation of L-AHA was analyzed in presence of the translation inhibitor Thiostrepton (10,138) (left panel) and absence of KsgA/Dim1 (right panel). Cells grown in Hv-min lacking methionine were pulse-labeled with 1 mM L-AHA for the indicated time point, except for the Thiostrepton experiments where cells were labeled for 3 h in presence of antibiotic. The extracted l-AHA-containing proteins were fluorescently labeled by click-chemistry with DBCO-Cy7 and separated by SDS-PAGE. Fluorescent signals were acquired on a Li-COR Odyssey platform. (D–F) Effect of KsgA/Dim1 on homeostasis of a subset of proteins in *H. volcanii* and *E. coli*. Volcano plot results of label-free proteomics analysis comparing (**D**) WT versus KsgA/Dim1 deletion strains, (**E**) reintegrated WT and KsgA E84A catalytic mutant in *H. volcanii*, and (**F**) WT vs Δ*ksgA/dim1* in *E. coli* are provided. The *P-*value cut-off for the up- (log_2_ FC > 0) and down-regulated (log_2_ FC < 0) proteins was set at *P* < 0.05, which is indicated by a dashed line in the plots. * Note that during data processing in Perseus, null or missing values were arbitrary imputed (see Materials and Methods), therefore the KsgA/Dim1 fold change in the knock out strains (null values) is arbitrary and is only provided to facilitate general analysis of the full dataset. ArlA1 (*HVO_1210*) = major archaeal flagellin precursor. (**G**) Functional classification of the differentially detected proteins by mass-spectrometry. (Archaeal) Clusters of orthologous genes (arCOGs/COGs) enrichment analysis derived from the mass-spectrometry analyses shown in (D)–(F) is provided. (ar)COG classes are as follows: J, translation, ribosomal structure and biogenesis; U, intracellular trafficking, secretion, and vesicular transport; F, nucleotide transport and metabolism; H, coenzyme transport and metabolism; O, post-translational modification, protein turnover, chaperones; Q, secondary metabolites biosynthesis, transport and catabolism; I, lipid transport and metabolism; V, defense mechanisms; E, amino acid transport and metabolism; C, energy production and conversion; L, replication, recombination and repair; T, signal transduction mechanisms; R, general function prediction only; D, cell cycle control, cell division, chromosome partitioning; P, inorganic ion transport and metabolism; K, transcription; M, cell wall/membrane/envelope biogenesis; G, carbohydrate transport and metabolism; S, function unknown; N, cell motility; X, mobilome. Contribution of each category is calculated in percentage and compared to the total background set. Enrichment analysis is based on the comparison of the total number of genes found in an (ar)COG category (rest, grey) and the number of genes that are significantly upregulated (red) or downregulated (blue). When applicable coloured arrows indicate that categories are significantly over- (pointing up) or under- (pointing down) represented in the upregulated (red) or downregulated (blue) genes (False discovery rate: 0.1). (**H**) Analysis of start codon usage distribution. Proteins identified by mass-spectrometry in (D–E) *H. volcanii* and (F) *E. coli* were plotted according to their relative level (*P-*value cut-off *P* < 0.05) and annotated start codon usage (AUG versus non-AUG).

To better understand the molecular basis of the phenotypes described above, and, moreover, since previous studies suggested that KsgA/Dim1 is required for translation initiation and fidelity (16,22,23,26), we aimed to determine the global impact of archaeal KsgA/Dim1 mutation on protein homeostasis. First, we monitored the effect of KsgA/Dim1 deletion on bulk translation, by performing a pulse-labelling experiment with the amino-acid analogue L-AHA for 30 min (∼12% of doubling time in these growth conditions) (70,72). The L-AHA-containing proteins were further fluorescently labeled by click-chemistry and analyzed by 1D and 2D SDS-PAGE (Figure 3 and Supplementary Figure S1). Within the time window analyzed, bulk translation activity of the KsgA/Dim1 deficient strain was not significantly affected compared to the parental wildtype strain (Figure 3C). In contrast, 2D gel analysis of the same samples revealed that the expression of a sub-set of proteins was perturbed in the absence of KsgA/Dim1 (Supplementary Figure S1).

To obtain a more comprehensive view on the different proteins affected by the absence of KsgA/Dim1, and in order to better define the molecular mechanisms underlying their translation regulation, we performed whole proteome label-free quantitative mass-spectrometry analysis, which allowed us to profile the relative expression level of >2300 individual proteins across various conditions (Figure 3D and E). Among the proteins identified in wildtype and the KsgA/Dim1 deletion cells only a restricted subset was significantly downregulated (∼9.2%) and to a smaller extend upregulated (∼2.2%) (threshold *P-*value < 0.5) (Figure 3D). Consistent with the motility phenotype described above (Figure 2), the major archaellin precursor ArlA1 (previously named FlgA1) (HVO_1210), a protein crucial for cell motility, was downregulated in the absence of KsgA/Dim1 (Figure 3D and E) (96,97,102,103). Quantitative RT-PCR analysis revealed that the mRNA encoding for the major archaellin precursor, ArlA1 (HVO_1210) (104,105), was expressed at similar levels in wildtype and KsgA/Dim1 deleted cells, indicating that the observed changes are at the translational/post-translational level (Supplementary Figure S1C).

In order to distinguish between different effects due to loss of KsgA or loss of 16S rRNA dimethylations, and to obtain further insights into the core KsgA/Dim1 regulatory mechanisms, we extended our proteomic analysis to cells expressing reintegrated wildtype KsgA/Dim1 and/or catalytic mutant (E84A) in the *H. volcanii* deletion background (Figure 3E). Moreover, to obtain an evolutionary perspective on the core regulated proteome across evolutionary divergent organisms, we additionally performed comparative label-free proteomic in wildtype, and KsgA/Dim1 deleted bacterial *E. coli* cells (Figure 3F).

Proteome analysis of *H*. *volcanii* reintegration strains proved unexpectedly challenging due to strong variability across the independent biological replicates most likely caused by independent reintegration events. This inconsistency may be explained by additional variability accumulated during the genetic manipulation procedure. Nevertheless, the major archaellin precursor ArlA1 is among the most decreased proteins (Figure 3E). This observation is in agreement with the motility phenotype observed in the reintegrated KsgA/Dim1 catalytic mutant strain (Figure 2). Additional proteomic analysis of bacterial (*E. coli*) KsgA/Dim1 deletion strains suggested that down- and upregulation of a restricted subset of the proteome does occur to a similar extent in this evolutionary divergent organism (Figure 3F).

We next performed a functional clustering analysis of the up- and downregulated proteins, however, we could not derive any obvious specific communalities that may account for the specific regulation of these open reading frames (Figure 3G). Moreover, we could not determine any obvious common core functional category overlap and/or homologies between the set of proteins regulated in *H. volcanii* and *E. coli* (Figure 3G). It should also be noted, that biological replicates of bacterial KsgA/Dim1 deletion strains (two independent deletions) obtained from the Keio collection (106), showed significant variability, affecting the overall statistical confidence of the results (Figure 3F) and rendering meaningful comparative analysis difficult.

Despite these variabilities, our results suggest that the molecular basis for the observed differential expression of a subset of proteins may be related to intrinsic common mRNA properties. In agreement with this conclusion, a previous study in bacteria, indicated that elevated level of translation initiation at non-AUG start codon occurs in KsgA/Dim1 deficient cells (24). However, since this data was based on analyses of a gene-reporter system, we examined the distribution of non-AUG start codons among our differentially expressed proteins on a more global scale. In agreement with previous published data (24), up-regulated proteins in *E. coli* and *H. volcanii* KsgA/Dim1 deletion strains were enriched for mRNAs utilizing non-AUG as a start codon (Figure 3H and Supplementary Figure S1D). In contrast, the subset of downregulated proteins was predominantly encoded by mRNAs containing canonical AUG as a start codon. However, since not all AUG-start codon containing proteins were downregulated, it is likely that additional layer(s) of regulation differentially affecting these proteins may exist (Figure 3H and Supplementary Figure S1D). The possible functional relevance of this finding will be analyzed in subsequent studies.

In summary, our results suggest that KsgA/Dim1 influences translation efficiency of a subset of mRNAs, which are, to some extent, organism specific. Remarkably, proteins with non-canonical AUG as start codon are more susceptible to be upregulated in the absence of KsgA/Dim1, across evolutionary distant organisms (24). Finally, the molecular mechanisms underlying the down regulation of a subset of proteins is not fully understood but may be due to differential start-codon selection efficiency.

### Analysis of KsgA/Dim1-dependent rRNA modifications in model archaea

**Figure 4.**
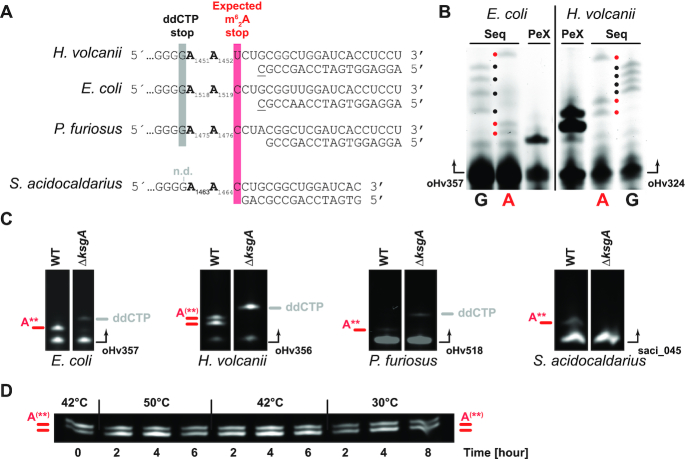
Analysis of KsgA/Dim1-dependent 16S rRNA modifications-status in representative model archaea. (**A**) Principle of KsgA/Dim1-dependent 16S rRNA modifications detection by primer extension. 16S rRNA 3′end sequences and the respective primers used for the indicated organisms are depicted. Expected primer extension stop upstream of the first encountered dimethylated adenosine and the first ddCTP induced primer extension stop (when applicable) at the first encountered guanosine downstream of the two modified adenosines indicative of read-through are displayed. Note that primer extension for *S. acidocaldarius* were not performed in presence of ddCTP (n.d.). (**B**) KsgA/Dim1-dependent 16S rRNA modifications-status analysis in *H. volcanii*. Exemplary results of primer extension (PeX) analysis performed on *E. coli* and *H. volcanii* 16S rRNA. Sequencing ladder (Seq) enabling the relative positioning of adenosine (A, red dot) and guanosine (G, black dot) are provided. Primers are indicated by arrows. (**C**) Verification of KsgA/Dim1 deletion strains by primer extension analysis. Primer extension analysis of parental strain and KsgA/Dim1 deleted strain in *E. coli*, *H. volcanii*, *P. furiosus* and *S. acidocaldarius* was performed in presence of dNTPs, except that for *E. coli*, *H. volcanii* and *P. furiosus* where ddCTP as a measure of readthrough was used instead of dCTP. Primers are indicated by arrows. Position of primer extension stops prior to the dimethylated adenosine are indicated in red, and ddCTP-induced primer extension stops indicative of read-through are, when applicable, indicated in grey. Primers are indicated by arrows. (**D**) KsgA/Dim1-dependent 16S rRNA modifications-status of *H. volcanii* cells examined in different growth conditions. Wildtype cells grown at 42°C (*t* = 0) were diluted and further grown at the indicated temperature for the indicated time. KsgA/Dim1-dependent 16S rRNA modifications-status was determined by primer extension as described above.

As indicated earlier, KsgA/Dim1 modifies the two universally conserved adenosine residues located within h45 at the 3′end of the SSU rRNA (see Introduction). In order to confirm the absence of KsgA/Dim1 catalytic activity in the deleted strains and obtain additional mechanistic insights into the KsgA/Dim1-dependent rRNA modification process in archaea, we used primer extension analysis as a well-established read-out procedure to reveal the presence of the two dimethyl adenosines located at the 3′end of the 16S rRNA (A_1518_ and A_1519,_*E. coli* numbering) (Figure 4). As previously demonstrated, reverse transcription reaction is fully inhibited by the presence of m^6^_2_A modifications, thereby generating a primer extension stop prior to the first encountered dimethylated adenosine, indicative of full dimethylation at A_1519_ (*E. coli* numbering) (16,19,23,37) (Figure 4A). In agreement, reverse transcription performed on 16S rRNA derived from the model bacteria *E. coli* was inhibited by the presence of the first encountered m^6^_2_A modification (Figure 4B and C). Similarly, primer extension performed on 16S rRNA extracted from *S. acidocaldarius*, and *P. furiosus* was also inhibited by the presence of the rRNA modifications at the first encountered dimethyl adenosine (A_1464_, *S. acidocaldarius* numbering) (Figure 4C). In contrast, primer extensions performed on 16S rRNA derived from *H. volcanii* was not fully inhibited at the expected first encountered modified adenosine but revealed two reproducible consecutive primer extension stops corresponding to the two universally conserved adenosines (A_1451_ and A_1452_, *H. volcanii* numbering) (Figure 4B and C). This specific pattern suggests an incomplete reverse transcription inhibition at the two putatively modified adenosines in *H. volcanii* and was reproducibly observed under different growth conditions (Figure 4D). Finally, in all conditions analyzed, primer extension was inhibited by the presence of KsgA/Dim1-dependent modifications as indicated by the lack of efficient read-through beyond the two adenosine residues in wildtype conditions (Figure 4).

However, and in contrast to the other organisms analyzed, the unique presence of two consecutive primer extension stops in *H. volcanii* indicates that the individual 16S rRNA positions are not fully modified and suggests that the KsgA/Dim1-dependent modifications are limited in *H. volcanii*. Accordingly, we hypothesized that this peculiar modification-status is (i) either dependent on limiting amounts and/or activity of the KsgA/Dim1 methyl transferase *in vivo* and/or (ii) due to reduced efficiency of h45 to be used as substrate. To differentiate between these possibilities, and to analyze the molecular principles regulating KsgA/Dim1-dependent modifications, we went on to examine the effect of excess KsgA/Dim1 to the overall modification’ equilibrium in *H. volcanii*.

### Additional *Haloferax volcanii* KsgA/Dim1 does not affect the apparent modifications equilibrium *in vivo* and *in vitro*

We hypothesized that if KsgA/Dim1 (*Hv*_KsgA/Dim1) catalytic activity and/or levels are limited in *H. volcanii*, providing additional quantities of *Hv*_KsgA/Dim1 *in vivo* or *in vitro* could be sufficient to alter the overall modification equilibrium and reach near completion as generally observed in other organisms.

**Figure 5.**
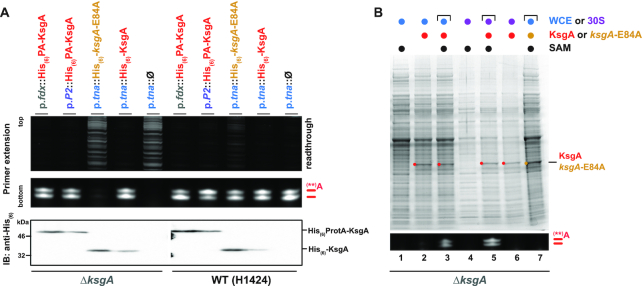
KsgA/Dim1-dependent modifications-status is not influenced by the relative amount of KsgA/Dim1. (**A**) Effect of KsgA/Dim1 overexpression on 16S rRNA modifications-status in *H. volcanii*. 16S rRNA modifications-status analysis of strains deleted for KsgA/Dim1 (complementation) or WT H1424 (additive expression) (left and right panel, respectively), transformed with the indicated plasmids containing N-terminally-tagged [histidine hexamer (His_6_) or Protein-A histidine hexamer (His_6_PA)] KsgA/Dim1 variants under the control of the constitutive ferredoxin (p.*fdx*) (108) or *Halobacterium cutirubrum* rRNA P2 (p.*P2*) (107,109) promoters grown in Hv-Ca^+^, or the tryptophan inducible tryptophanase (p.*tna*) (98) promoter grown in Hv-Ca^+^ supplemented with 0.5 mM tryptophan is provided. Upper panel displays primer extension read-through signals visible in the upper part of the denaturing urea–TBE–PAGE. Middle panel shows the KsgA/Dim1-dependent modifications. Verification of additional expression of the plasmid encoded KsgA/Dim1 variants by immunoblot using a probe directed against the histidine hexamer is provided in the lower panel. (**B**) *In vitro* reconstitution of KsgA/Dim1-dependent 16S rRNA modifications in *H. volcanii*. Whole cell extract (WCE) or isolated small ribosomal subunit (30S) from Δ*ksgA/dim1 H. volcanii* strain were incubated in presence of excess of recombinantly purified wildtype KsgA or catalytically inactive KsgA (*ksgA*-E84A) and/or 1 mM S-adenosyl-Methionine (SAM) for 1 h at 37°C. Ribosomal RNA modifications were monitored by primer extension analysis (lower panel). Upper panel: Coomassie staining of the respective reaction mixture separated by SDS-PAGE is shown. Recombinant KsgA wildtype and catalytic mutant (*ksgA*-E84A) are indicated.

To test this possibility, we additively co-expressed wildtype *Hv*_KsgA/Dim1 and the catalytic mutant (E84A) in wildtype *H. volcanii* cells using strong promoters of different relative strength (98,99,107–109), and analyzed the respective KsgA/Dim1-dependent modification patterns by primer extension. To confirm that the plasmid-expressed wildtype KsgA/Dim1 is functionally active, we first performed functional complementation and co-sedimentation analyses with the small ribosomal subunits. As shown in Figure 5, plasmid-based expression of wildtype KsgA/Dim1 in a KsgA/Dim1 deletion background is sufficient to restore the *H. volcanii* specific modification pattern to wildtype levels (Figure 5A, left panel). Moreover, a fraction of KsgA/Dim1 expressed under similar conditions specifically co-sedimented with its substrate, the small ribosomal subunit (Supplementary Figure S2). Moreover, a significant fraction of free KsgA/Dim1 (non-ribosomal subunit-associated) was also observed in these conditions.

Thus, having demonstrated the functional capability of these constructs (see also Figure 2), we have investigated their effects when additionally expressed in a wildtype context. As shown in Figure 5, the expression of wildtype *Hv*_KsgA/Dim1 did not significantly change the steady-state level and pattern of the 16S rRNA modifications. In contrast, co-expression of the *Hv*_KsgA/Dim1 catalytic mutant (E84A) decreased the overall amount of modified rRNA molecules, as indicated by an increase in primer-extension read-through across the modified adenosine residues, without significantly modifying the primer-extension steady-state pattern (Figure 5A).

In absence of additional analytical tools, the required levels of *Hv*_KsgA/Dim1 sufficient to enable the establishment of an alternative steady-state modification pattern *in vivo* is difficult to predict. Therefore, we also *in vitro* reconstituted KsgA/Dim1-dependent modification patterns using recombinant *Hv*_KsgA/Dim1 and partially purified native SSU from *H. volcanii*. As shown in Figure 5, we could reconstitute the KsgA/Dim1-dependent modification pattern *in vitro* using either whole-cell extract or partially purified SSU from cells lacking the KsgA/Dim1 activity. In agreement with our *in vivo* results, and despite excess addition (up to 90-fold excess) of recombinant protein (see Figure 5B, upper panel), the *Hv*_KsgA/Dim1-dependent modifications *in vitro* were also incomplete, independent of the conditions and batch of purification used. Although, our experimental set-up do not allow us to quantitatively determine the amounts of active KsgA/Dim1 added in our *in vitro* and *in vivo* experiments we concluded that the KsgA/Dim1 activity is unlikely to be the main limiting molecular determinant responsible for the incomplete and/or heterogenous 16S rRNA modification-status observed in *H. volcanii*.

### A conserved h45 sequence/structure variation and the limited KsgA/Dim1-dependent modification is shared among phylogenetically related archaea

**Figure 6.**
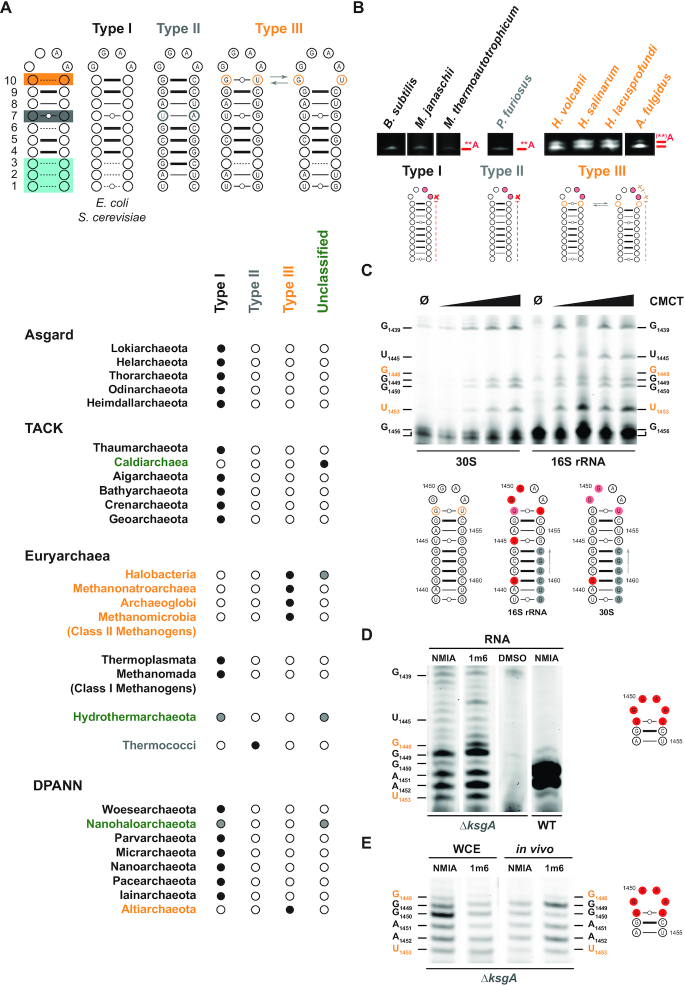
Conserved RNA sequence/structure and KsgA/Dim1-dependent modifications-status is shared by phylogenetically related archaea. (**A**) 16S rRNA helix 45 secondary structure prediction diversity across archaea. Archaea were classified into three major groups (type I, II and III) based on h45 sequence alignment and structure prediction of representative members of these groups. Base-pairing numbering (1-10) was annotated from the base of the h45 stem. When invariant the corresponding nucleotides and nature of the base pairing (G-C larger line, A-U thinner line or G•U dotted thinner line) are provided. Base pairing variability is indicated by dashed thinner line. An average archaeal h45 structure based on all the compiled sequence (see material and methods) is depicted (upper left). Note that the first six nucleotides forming the first 3 base-pairs (indicated in blue) are relatively diverse in comparison to the almost invariant base-pairs at position 4–6 and 8/9 across archaea. Variability at G•U base-pairing at position 7 observed in type II and at position 10 specific to the type III group are indicated in grey and orange, respectively. Repartition of the different h45 type across archaea are provided based on recent archaeal classification (110–112). Exemplary sequences and structure prediction for diverse representing archaeal organisms are provided in Supplementary in Figure S3. Black and white dot indicate presence and absence, respectively. Grey dot indicates presence of at least two h45 structure types. Note that the unclassified h45 variation in Halobacteria is restricted to the *Haloarcula* genus. (**B**) Common KsgA/Dim1-dependent 16S rRNA modifications-status across phylogenetically related archaea. Analysis of KsgA/Dim1-dependent dimethylation was performed in representative type I, II and III organisms. The expected/obtained dimethylation-induced primer extension stop(s) prior to the respective modified adenosines are indicated in red. Schematic representation based of h45 secondary structure prediction in the different groups is depicted (lower panel). (C–E) *In vitro* and *in vivo* analysis of helix 45 structural features in *H. volcanii*. Nucleotide reactivity to chemical footprinting reagents was performed *in vitro* with CMCT (**C**) and *in vitro* and *in vivo* with the indicated SHAPE reagents (D, E). Relative nucleotide reactivity, as obtained from increased primer extension inhibition, is indicated in red on the 2D RNA prediction model. In grey are nucleotides covered by the primer used. The nucleotides involved in wobble base-pairing ending the stem at position 10 (w10) are marked in orange. *H. volcanii* numbering is used. RNA shape (**D**) was performed on extracted RNA, WCE SHAPE (**E**) on whole cell extract and *in vivo* SHAPE by addition of shape reagent in the culture media.

Next, we focused on structural/sequence variation in the 16S rRNA as possible reason for the incomplete KsgA/Dim1-dependent modifications in *H. volcanii*. To this end, we collected 16S rRNA sequences representative of most of the main archaeal phyla (87,110–112). The compiled sequences were aligned, and the h45 sequences, which encompass the corresponding KsgA/Dim1 substrate, were extracted, grouped according to sequence similarity, and representative sequences were used for RNA structure predictions (89) (Figure 6A and Supplementary Figure S3). Simultaneously, to get a general overview across the tree of life, we performed h45 structure predictions and classification of bacterial and eukaryotic cytoplasmic SSU (Supplementary Figure S4).

Our structure-based h45 classification enabled us to group most of the archaeal h45 sequences into three major groups. In contrast, few archaea showed divergent h45 structure predictions which were apparently confined to the individual members analyzed and/or restricted to few archaeal organisms (Figure 6A and Supplementary Figure S3). Interestingly, most archaea belong to one group – hereafter called h45-type I—displaying a similar 2D RNA structure prediction despite sequence diversity (Figure 6A and Supplementary Figure S3). The h45-type I group contains phylogenetically diverse archaea, (e.g. TACK and Asgard Superphylum, Methanogens class I and many more) and is structurally characterized by the presence of a G–C base pairing closing the modified GGAA tetraloop at position 10 (relative to stem base) and the presence of wobble base pairing (G•U) at position 7 within the stem of h45 (Figure 6A and Supplementary Figure S3). This structural signature is also commonly observed in most bacteria and eukaryotes and may represent the archetype h45 structure (Supplementary Figure S4). The second group – hereafter called h45-type II—exclusively contains representative organisms of the Thermococci class and is absent in bacteria and restricted to few eukaryotes (Supplementary Figure S4). The h45-type II group is structurally characterized by the presence of a G–C base pairing closing the modified GGAA tetraloop at position 10 and the presence of a A-U base pairing instead of G•U at position 7 in the stem of h45 (Figure 6A and Supplementary Figure S3). The third group—hereafter called h45-type III—is predominantly found in archaea and is mostly restricted to organisms sharing a common phylogenetic ancestor (Archaeoglobi, Methanogens class II, most Halobacteria) (110,111) (Figure 6A and Supplementary Figure S3). The h45-type III group is structurally characterized by the presence of a G•U wobble instead of the common G-C base pairing ‘closing’ the modified GGAA tetraloop at position 10 and the regular presence of a wobble base pairing (G•U) at position 7 within the stem of h45 (Figure 6A and Supplementary Figure S3).

G•U base pairing closing RNA stem may have higher probability to adopt a partial open conformation (89,113), thereby possibly increasing the structural dynamics of h45 in this group of organisms to which *H. volcanii* belongs. Accordingly, if the RNA structure/sequence shared within this group contributes to the KsgA/Dim1-dependent modification variation observed in *H. volcanii*, the h45 modification pattern should be qualitatively similar in these phylogenetically related archaea. To test this hypothesis, we analyzed the h45 modification-status in selected cultivatable representative archaea belonging to the type I-III groups. As shown in Figure 6, all primer extension reactions, using diverse source of type III organisms (here Halobacteria and Archaeoglobi), showed similar modification pattern as was observed for *H. volcanii* (Figure 6B), whereas type I and II exhibited genuine modification-status as previously observed in most bacterial and eukaryotic analyzed to date. Taken together, our *in silico* RNA structure prediction and primer extension analyses suggest that the G•U base-pairing terminating the h45 stem may contribute to the observed divergent KsgA/Dim1-dependent modification pattern in *H. volcanii* and a subgroup of archaea.

To further determine the consequences of the G•U wobble on the h45 secondary structure and to confirm our *in-silico* RNA structure prediction, we performed RNA chemical structure probing experiments using CMCT *in vitro*, which primarily modifies single-stranded U and less efficiently unpaired G, and SHAPE-reagents *in vivo*, to analyze local nucleotide flexibility. Both CMCT- and SHAPE-dependent modifications inhibit reverse transcription and, accordingly, the relative nucleotide reactivity can be determined by primer extension analysis (90,114,115). As shown in Figure 6, G_1448_ and U_1453_, presumably engaged in a more dynamic wobble base pairing at the tip of the stem of h45, were both highly reactive to CMCT *in vitro*, independent of their molecular context (naked RNA or protein-bound), indicating in agreement with our 2D RNA prediction, that the h45 terminal G_1448_/U_1453_ pair adopts an open/dynamic conformation (Figure 6C). Furthermore, *in vivo* SHAPE analysis of wildtype and KsgA/Dim1 deleted strains, also confirmed the higher conformational flexibility of the h45 terminal G_1448_/U_1453_ (Figure 6D and E).

Taken together, our data suggest that the h45-type III group adopts a different structural architecture which may influence the overall KsgA/Dim1-dependent modification pattern of the 16S rRNA.

### RNA sequence/structure variation is a key determinant for limited KsgA/Dim1-dependent modifications in phylogenetically related archaea

**Figure 7.**
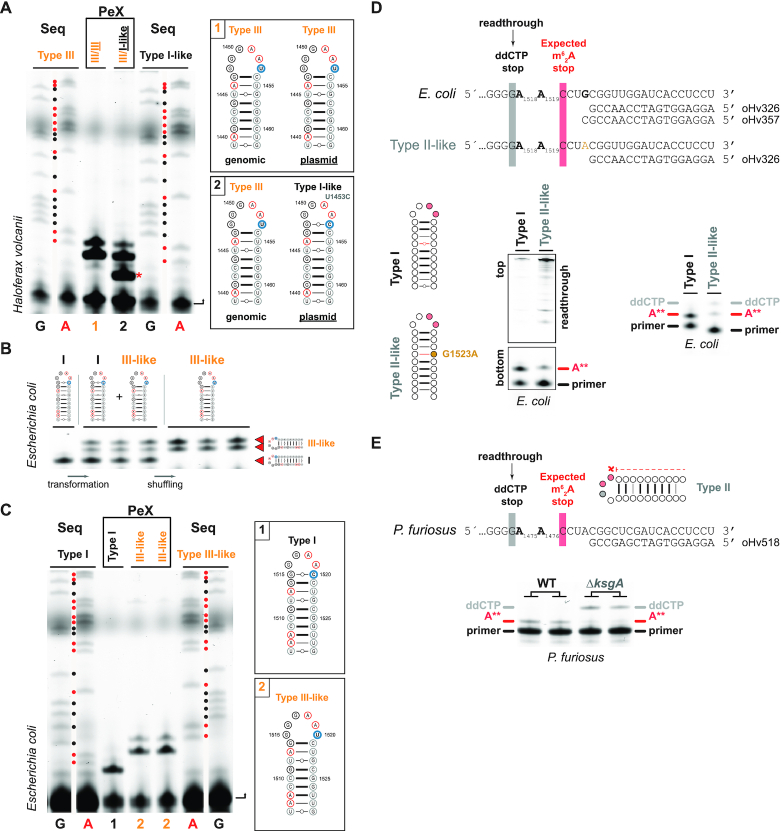
Molecular determinants controlling the diversity and adaptation of KsgA/Dim1-dependent modifications-status across archaea. (**A**) Analysis of KsgA/Dim1-dependent 16S rRNA modifications-status of *H. volcanii* cells co-expressing type I-like h45. KsgA/Dim1-dependent 16S rRNA modifications-status of wildtype cells co-expressing type III or type I-like rRNA variants were analyzed by primer extension. Exemplary results of primer extension (PeX) are provided. Sequencing ladder (Seq) for the relative positioning of adenosine (A, red dot) and guanosine (G, black dot) are shown. Primers are indicated by arrows. Note the template-dependent size migration differences observed for the first incorporated nucleotides for both primer extension analysis and sequencing reference of type III and type I-like. Plasmid encoded rDNA are underlined. The corresponding RNA secondary structure and nucleotide exchange (circled in blue) are provided on the right side. Red asterisk (*) indicates the unique expected and obtained primer extension stop induced by complete dimethylation of type I-like derived 16S rRNA. (**B**, **C**) Analysis of KsgA/Dim1-dependent 16S rRNA modifications-status in *E. coli* cells expressing type III-like h45. (B) KsgA/Dim1-dependent 16S rRNA modification status of rDNA-shuffle strain, prior to transformation (left side), co-expressing endogenous type I and plasmid-based type IIII-like rRNA prior to shuffling, and only expressing type III-like rRNA variants after shuffling (right side) were analyzed by primer extension. Exemplary results of primer extension (PeX) analysis for individual transformants before and after-shuffling the endogenous wildtype rDNA plasmids are provided. Dimethylation-dependent primer extension stops are indicated by red arrows. (**C**) Analysis of KsgA/Dim1-dependent 16S rRNA modifications-status in *E. coli* cells solely expressing type III-like h45. KsgA/Dim1-dependent 16S rRNA modification status of cells solely expressing type I or type III-like h45 were analyzed as in (B). Exemplary results of primer extension (PeX) are provided. Sequencing ladder (Seq) for the relative positioning of adenosine (A, red dot) and guanosine (G, black dot) are shown. Primers are indicated by arrows. Note the template-dependent size migration differences observed for the first incorporated nucleotides for both primer extension analysis and sequencing references of type I and type III-like. The corresponding RNA secondary structure and exchanged nucleotide (circled in blue) are provided on the right side. (**D**) Analysis of KsgA/Dim1-dependent 16S rRNA modifications-status in *E. coli* cells expressing type II-like h45. WT and type II-like *E. coli* 16S rRNA 3′end sequence and the primers used for primer extension are depicted. The expected primer extension stop upstream of the first encountered dimethylated adenosine (A_1519_ – *E. coli* numbering) and the first ddCTP induced primer extension stop at the first encountered guanosine (G_1517_–*E. coli* numbering) downstream of the two modified adenosines indicative of read-through are indicated. KsgA/Dim1-dependent 16S rRNA modification status of *E. coli* cells solely expressing type I or type II-like h45 were analyzed in presence of dNTPs (middle panel) or in presence of ddCTP as described above (right panel). Primer extension read-through were visualized either on the top part of the Urea-TBE-PA gels (dNTPs, middle panel) or at the indicated ddCTP-induced stop (right panel). Note that different primers (type I: oHv357 and type II-like: oHv326) where required to allow read-through analysis with ddCTP. The dimethylation-induced primer extension stop prior to the first encountered modified adenosine is indicated in red and ddCTP-induced primer extension stops indicative of read-through are indicated in grey. The corresponding RNA secondary structure and nucleotide exchange (in orange) are provided on the left side. (**E**) *P. furiosus* KsgA/Dim1-dependent modifications-status analysis. *P. furiosus* 16S rRNA 3′end sequence and the primer (oHv518) used for primer extension are depicted. The expected primer extension stop upstream of the first encountered dimethylated adenosine (A_1476_ – *P. furiosus* numbering) and the first ddCTP induced primer extension stop at the first encountered guanosine (G_1474_ – *P. furiosus* numbering) downstream of the two modified adenosines indicative of read-through are indicated. Primer extension analysis of *P. furiosus* parental and Δ*ksgA/dim1* strain was performed in presence of dNTPs, except that dCTP was replaced by ddCTP to score primer extension read-through downstream of the targeted adenosines. Position of primer extension stops prior to the dimethylated adenosine are indicated in red, and ddCTP-induced primer extension stops indicative of read-through are indicated in grey.

To further support our findings, we took advantage of our recently described plasmid-based *cis*-acting element reporter system (10) and analyzed the induced rRNA modification-status after mutating h45 in *H. volcanii*. We generated a G_1448_•U_1453_ to G_1448_-C_1453_ (h45-type I-like) mutant and co-expressed this rRNA variant in addition to the endogenous type III rRNA in *H. volcanii*. During our analysis, we also noticed the presence of a sequence-specific shift in the migration behavior of the very first nucleotides in our control ddNTP sequencing references (Figure 7A). Therefore, and in the absence of an rDNA shuffling system in *H. volcanii*, we used this difference in migration behavior to explore the modification pattern of plasmid- and genomic-derived 16S rRNA in *H. volcanii*. As shown in Figure 7, expression of plasmid derived type I-like h45 16S rRNA generated an additional, faster migrating, primer extension stop presumably corresponding to the plasmid-derived first encountered dimethylated adenosine (indicated by an asterisk in Figure 7A). Notably, no additional bands were observed at the expected height of the plasmid-derived h45 second dimethylated adenosine (Figure 7A). While, these results are only suggestive of completed modifications of type I-like 16S rRNA in *H. volcanii*, the absence of an rDNA knock-out system in *H. volcanii* prevented a more precise analysis of the 16S rRNA modification status of a pure type I-like h45 population.

To circumvent this limitation and to further ascertain the role of the terminal G•U base pairing at the tip of h45 stem in establishing alternative KsgA/Dim1 modification patterns, we made use of the well-established rDNA shuffling genetic system available in *E. coli* (91–93). Using this system, we analyzed the KsgA/Dim1-dependent modifications status of type III-like h45 *E. coli* 16S rRNA variant (G_1515_–C_1520_ to G_1515_•U_1520_) (116). After transformation and shuffling out the plasmid-borne type I wildtype rDNA, we determined the h45 KsgA/Dim1-dependent modification status of cells solely expressing the corresponding 16S rRNA type III-like variant (Figure 7B and C). In addition, we also analyzed cells co-expressing native type I h45 and episomal type III-like h45 rDNA variant for comparison to the rDNA co-expression situation in *H. volcanii* (Figure 7A). As depicted in Figure 7, the KsgA/Dim1-dependent modification pattern in *E. coli* cells expressing type III-like h45 were akin to the one observed for native type III h45 in *H. volcanii* (compare Figure 7A–C). Similar results were also obtained for *E. coli* and *H. volcanii* cells co-expressing h45 type I/type III rDNA variants (compare Figure 7A–C).

Taken together, these results strongly support the findings that the terminal G•U base pairing at the tip of h45 stem is sufficient to alter the KsgA/Dim1-dependent RNA modification patterns encountered in a subset of phylogenetically related archaea, suggesting a model in which the intrinsic h45 structure determines its modification efficiency in a closely related group of archaea.

### Additional adaptation of KsgA/Dim1-dependent modifications in Thermococci

As indicated above, members of the Thermococci class form a specific group characterized by the absence of the G•U and presence of A-U base pairing at position 7 within the h45 stem (Figure 6 and Supplementary Figure S3). A previous study analyzing the effect of h45 mutations for the efficiency of the KsgA/Dim1-dependent modifications demonstrated that reconstituted bacterial ribosomal subunits carrying a U_1512_•G_1523_ to U_1512_–A_1523_ exchange at position 7 are poor substrates for recombinant *E. coli* KsgA/Dim1 *in vitro* (∼25% modifications) (117). These results raised several questions regarding the efficiency of the KsgA/Dim1-dependent reaction and how m^6^A modification maybe distributed on type II (-like) h45 substrate *in vivo*.

First, we analyzed the *in vivo* effects on KsgA/Dim1-dependent modifications of type II-like substrate in *E. coli*. Similar to the strategy described above, we generated a strain solely expressing a type II-like h45 variant (G_1523_ to A_1523,_*E. coli* numbering) mimicking the *P. furiosus in vivo* state at this position (Figure 7D). Using a similar primer extension set-up described above, we determined that the type II-like h45 16S rRNA was less efficiently modified in *E. coli in vivo*, as indicated by the presence of increased read-through (ddCTP induced stop at G_1517_) and relative decrease of primer extension stop at the first encountered adenosine (A_1519_) compared to the wildtype native substrate (Figure 7D).

According to these results, we next analyzed the modification efficiency of the KsgA/Dim1-dependent modifications in *P. furiosus* by evaluating the amounts of read-through using primer extension in the presence of ddCTP (Figure 7E). Under these experimental conditions, any reverse transcriptase read-through, which is indicative of hypo-/non-modified rRNA population, will generate a chain-termination stop at the first encountered guanosine (G_1474,_ *P. furiosus* numbering) in the sequence downstream of the two expected modified adenosines (A_1475_ and A_1476_). In contrast to our *in vivo* analysis of type II-like h45 variant in bacteria, no significant primer extension stop was observed downstream of the modified adenosines in *P. furiosus*, and a single primer extension stop was observed prior to the first encountered modified adenosine residue (Figure 7D). These results suggest that dimethylation modification at the first encountered adenosine targets the majority of 16S rRNA molecules and is overall likely to be unperturbed in *P. furiosus*.

Overall, these results indicate the presence of a *Pfu*_KsgA/Dim1 functional adaptation to fully modified type II h45 substrate in *P. furiosus*, a feature which is apparently absent or less efficient in *E. coli*. Moreover, although less efficient, the modifications at the two adenosines appear to be complete, however, only on a limited subset of 16S rRNA molecules in *E. coli*. The molecular basis for the observed adaptation of KsgA/Dim1-dependent modification in *P. furiosus* is currently unknown and will be the focus of subsequent investigations.

### *In vitro* reconstitution of archaeal KsgA/Dim1 release from its small ribosomal subunit substrate

In contrast to the so far deduced KsgA/Dim1 release model from its substrate, the results presented above suggests that completion of h45 modifications is not a necessary step to induce KsgA/Dim1 release from the nascent SSU. Rather, our observations indicate that the h45 intrinsic properties and accompanying folding changes during the modifications process may affect the binding affinity landscape of KsgA/Dim1. Whether other factors cooperatively participate in the KsgA/Dim1 release process in archaea, similar to Fap7 in the yeast *S. cerevisiae* (33), is so far unknown.

**Figure 8.**
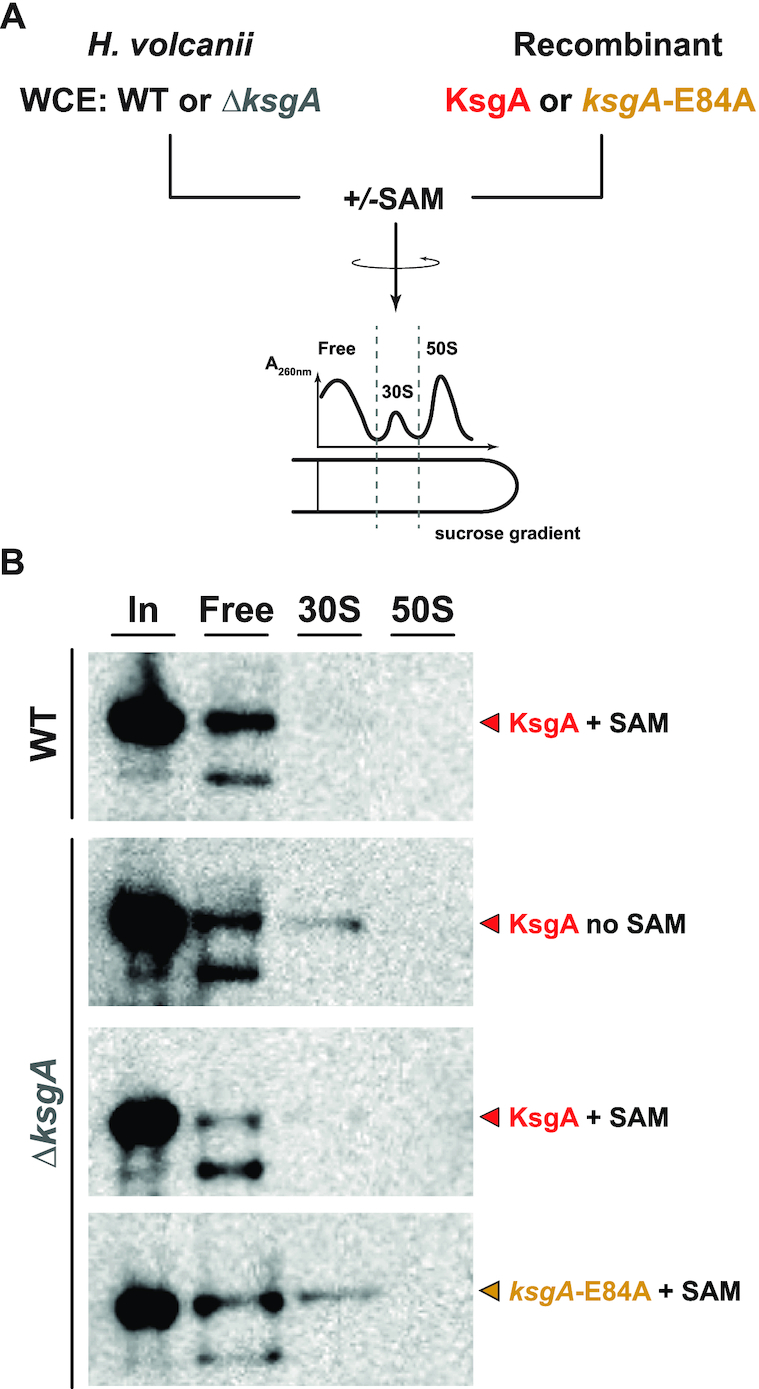
*In vitro* reconstitution of *H. volcanii* KsgA/Dim1 binding/release from its small ribosomal subunit substrate. (**A**) Experimental workflow. Whole cell extract (WCE) from wildtype (H26) or Δ*ksgA/dim1 H. volcanii* strain was incubated in presence of recombinantly purified wildtype KsgA or catalytically impaired KsgA (*ksgA*-E84A) and/or 0.5 mM S-adenosyl-Methionine (SAM) for 30 min at 42°C The reactions were separated on sucrose gradient by ultracentrifugation. Fractions corresponding to the non-ribosomal subunit associated (Free), 30S and 50S pool were collected and analysed by Western blotting. (**B**) Western-blot analysis of co-sedimentation behavior of recombinant KsgA/Dim1. Association of KsgA/Dim1/*ksgA*-E84A to its 30S substrate was monitored by immunoassay using a probe directed to the N-terminal hexa-histidine tag carried by the recombinant KsgA/Dim1/*ksgA*-E84A.

To gain a better understanding of the molecular requirements for archaeal KsgA/Dim1 release from the SSU, we have biochemically reconstituted this event *in vitro*. To analyze KsgA/Dim1 release from the SSU *in vitro*, we used recombinant *Hv*_KsgA/Dim1, and whole-cell extract (WCE) of a KsgA/Dim1 deletion strain in conditions enabling 16S rRNA modifications as described above (Figure 5). Recombinant wildtype and catalytic mutant (E84A) KsgA/Dim1 proteins were incubated with WCE in the presence or absence of S-adenosyl-methionine (SAM). The resulting KsgA/Dim1::30S complex formation was then monitored by co-sedimentation analysis on a sucrose gradient and detection of co-migrating KsgA/Dim1 (Figure 8A). As shown in Figure 8, in the absence of SAM, a larger fraction of wildtype KsgA/Dim1 was associated with the 30S SSU, while, in comparison, most wildtype KsgA/Dim1 was not associated with the SSU after completion of rRNA modifications in the presence of SAM, or if endogenously modified SSU derived from wildtype cells was used. In contrast, the catalytic KsgA/Dim1 mutant protein remained associated with the 30S particle independent of the presence or absence of SAM (Figure 8B).

Taken together these results provide additional insights into the molecular determinants influencing archaeal KsgA/Dim1 release competence (see Discussion below), and suggest that *Hv*_KsgA/Dim1 release is catalytic activity-, SAM- and h45-methylation-dependent, but appears to be independent of complete h45-methylation. Whether additional factors cooperatively participate in archaeal KsgA/Dim1-dependent release remains to be investigated.

## DISCUSSION

While bacterial and eukaryotic ribosome biogenesis have been extensively studied over the past decades, the archaeal ribosome biogenesis pathway still remains to be fully explored (6,118,119). Here, we present a functional characterization of the archaeal KsgA/Dim1 dimethyl transferase homolog. While KsgA/Dim1 functions have been largely studied in both bacterial and eukaryotic organisms (30,37,39,44,48), whether archaeal KsgA/Dim1 fulfils its function in a very similar fashion *in vivo* had not been previously addressed.

### *In vivo* function of KsgA/Dim1 in archaea

Using the advantage of now well-established archaeal genetic systems, we have performed gene deletion and functional characterization of the archaeal KsgA/Dim1 homolog in evolutionary divergent archaea. Remarkably, our study reveals that, similar to bacterial KsgA/Dim1, archaeal KsgA/Dim1 is not essential in three distantly related archaea. These results are reminiscent of those obtained for two other archaeal ribosome biogenesis factors, Rio1 and Rio2 (11,120). Previous analyses showed that neither Rio1 nor Rio2 are essential for the growth of *H. volcanii* and *S. acidocaldarius* in laboratory conditions (11,120). Although, *H. volcanii* KsgA/Dim1 is not essential for cell viability, additional molecular characterization suggests that *Hv_*KsgA/Dim1 and/or its rRNA modification activity is beneficial for cellular adaptation. This is illustrated by changes (i) in cellular fitness and (ii) in cellular differentiation behavior (motility/adhesion). To some extent, these phenotypes also correlated with fluctuations in the steady-state level of key proteins involved in motility and general cellular metabolism homeostasis. How KsgA/Dim1 and its modifications may contribute to establishing these apparently specific changes remains to be fully determined. Previous studies, suggested a role of the KsgA/Dim1-dependent modifications to ensure the functional integrity of the SSU A- and P-site (24,27,28,31). Accordingly, absence of bacterial KsgA/Dim1 and its activity are associated to general translation initiation defects and decrease translation fidelity (24,26,121,122). Whereas these functional considerations could suggest a global impact of KsgA/Dim1 loss of function on cellular proteostasis, our analysis does not provide evidence for a pleiotropic effect on translation activity associated to the absence of KsgA/Dim1 and/or its catalytic activity. In fact, bulk and short-time protein synthesis analyses using BONCAT suggests on the one hand, no major effect on protein synthesis and stability, and on the other hand, only specific effects on a subset of the *H. volcanii* proteome. In agreement, label-free proteomics analysis suggested a minor contribution of *Hv*_KsgA/Dim1 to sustain global translation activity and points to more specific alteration in the steady-state level of a subset of proteins. The cellular impact of KsgA/Dim1 loss of function appears to be conserved in its principle, at least to some extent. In fact, analyses performed in the model bacterium *E. coli* [this study and (25)] provide a very similar picture in this organism, where no major effect on protein synthesis and stability, and only discrete effects on a subset of the *E. coli* proteome could be scored [this study and (25)]. This is in stark contrast to a recent analysis using human cell culture where a greater effect on proteostasis was observed (122). However, the use of overexpression of catalytic inactive KsgA/Dim1, in a haploinsufficient human KsgA/Dim1 background, may account for the exacerbating effect on cellular transcription/translation observed under these experimental conditions (122). Indeed, overexpression of catalytic inactive KsgA/Dim1 leads to dominant-negative effects on various aspects of prokaryotic cellular physiology [(16), this study see Figure 2]. Unfortunately, these and our studies failed so far to fully illuminate the underlying molecular mechanisms enabling the regulation of these functionally diverse proteins across evolutionary distant organisms. Whereas a previous study (24) and our data further suggest a possible role of start-codon selection in the regulation of the translation output of a subset of proteins, future studies focusing on detailed functional and cross-evolutionary analysis will be required to reveal the exact molecular mechanisms by which KsgA/Dim1-dependent modifications may impact cellular proteostasis.

### Influence of h45 structural/sequence features on KsgA/Dim1-dependent modifications

KsgA/Dim1 was shown to perform the addition of four methyl groups on every single SSU rRNA molecule on two universally conserved adenosines located within the terminal helix (h45) at the 3′end of the SSU rRNA (16,17,20,21). Previous studies have shown that KsgA/Dim1-dependent rRNA modifications occur in a pre-established RNP context (123) that might (i) stabilize KsgA/Dim1 binding to the nascent SSU, (ii) regulate substrate accessibility and (iii) consequently, regulate the timing of this rRNA modifications. In addition, KsgA/Dim1-dependent rRNA modifications occur apparently during a restricted time window, as further assembly of the bacterial specific r-protein bS21 in *E. coli* is incompatible with rRNA modification activity *in vitro* (123–126). Although the window of opportunity for the KsgA/Dim1-dependent modification seems to be similar between bacteria and yeast, i.e. during late steps of SSU maturation, recent functional and structural analyses of human pre-ribosomal subunits suggests a slightly different order of events (35–37), which may have emerged in response to additional unknown molecular constraints imposed to the human ribosome biogenesis pathway.

In contrast to most ribosome biogenesis factors, which are usually not well conserved throughout the different domains of life, KsgA/Dim1 is unique as it is maintained across the different domains of life, and until recently was considered to be universally conserved. However, KsgA/Dim1 is absent in the obligate symbiont archaeon *N. equitans* (and some additional nano-sized archaea), where its function is presumably substituted by the presence of unique RNA-guided rRNA modifications distributed across h45 (19). Moreover, organelles SSU synthesis and function has also been shown to be either independent of KsgA/Dim1 and/or show an unusual rRNA modification patterns (47,48). Although these peculiarities may represent special cases, these findings nevertheless imply that cellular dependency on KsgA/Dim1 function and KsgA/Dim1-dependent modifications might potentially represent an unexplored diversity across the tree of life.

In this study, we provide additional evidence for such natural variability and how it is controlled at the molecular level in archaea. Our analysis suggests, on the one hand, that the KsgA/Dim1-dependent modifications are limited in a subset of evolutionary related organisms (type III group), and on the other hand, that additional functional adaptation, presumably at the protein level, may have emerged to facilitate KsgA/Dim1-dependent rRNA modifications of structurally altered substrates (e.g. type II group).

A group of organisms which mostly emerged from a common phylogenetic ancestor (110,111), including the Archaeoglobi, Methanogens class II and most Halobacteria analyzed, are characterized by the presence of a C>U exchange, generating a G•U base pair at the tip of the stem of h45 and an altered KsgA/Dim1-dependent rRNA modifications pattern (h45 type-III). Using cross-species rDNA genetic manipulation and RNA structural analyses, we could show that the C>U exchange is sufficient to modify (i) the structural properties of h45 and (ii) the apparent KsgA/Dim1-dependent methylation status.

Previous studies suggested that the introduction of the KsgA/Dim1-dependent methylations facilitates destabilization of the GNRA tetraloop found at the tip of h45 (27,127–130). This destabilization and the resulting structural rearrangements lead to the outward rotation of GAA from the GGAA tetraloop, thereby enabling the formation of hydrogen bonding between h44 and h45, which in turn stabilizes incorporation of h45 in its 3D structural environment (27,28,31). According to this model, pre-destabilization events, that would be still compatible with the final 3D rearrangement, may alter the KsgA/Dim1-dependent rRNA modification profile encountered at h45. In agreement with this idea, our results provide additional support to this scenario and indicate that the necessary opening and destabilization of the helix/tetraloop by the introduction of additional methylations can be modified in an h45 conformation-dependent manner. Together these results unravel a substrate conformation-dependent modification, which may potentially contribute to KsgA/Dim1 release mechanism (see below).

Furthermore, a previous study (117) proposed that the h45 stem sequence/structural integrity is important for the efficiency of the KsgA/Dim1-dependent modifications. Interestingly, none of these mutations, with only one exception, are naturally occurring (117). In fact, h45 of the members of the Thermococci class is characterized by a G•U to G-C exchange at position 7 relative to the stem beginning (h45 type-II). According to this previous study, *in vitro* reconstituted SSU carrying such a variant was poorly modified (∼25%) by recombinant *E. coli* KsgA/Dim1 (117). This early observation raised the question of whether KsgA/Dim1-dependent rRNA modifications are also limited in Thermococci and in bacteria carrying this variant *in vivo*. To clarify this question, we have analyzed the KsgA/Dim1-dependent modification status in a Thermococci representative, *P. furiosus*, and in a genetically modified *E. coli* strain solely expressing this rRNA variation (type II-like h45). Our study further supports that the bacterial KsgA/Dim1 capacity to methylate a type II-like h45 *in vitro* and *in vivo* is restricted in the experimental conditions tested [(117), this study]. In contrast, and in agreement with recent studies, this naturally occurring variant is a genuine substrate for KsgA/Dim1-dependent modifications in Thermococci [(131–133), this study]. Together these results suggest the presence of, yet undefined, additional molecular adaptation(s) in the Thermococci class, enabling efficient accommodation of type II h45 as a substrate.

The peculiar KsgA/Dim1-dependent rRNA modification pattern observed in exemplary archaeal type III organisms implies that the h45 modifications at the target adenosines are incomplete and that a heterogenous SSU population might be generated. Although the read-out power of our assay to resolve the different possible rRNA modification combinations in these cellular contexts is limited, the presence of at least two main populations can be logically deduced. One portion of the rRNA molecules are expected to be either hypo- or non-modified, on the most 3′ adenosine, or a combination thereof, and fully modified on the most 5′ adenosine. In contrast, the additional portion of the rRNA molecules have dimethylated 3′ adenosine and the 5′ adenosine may adopt a fully, partially or a non-modified status, or a combination thereof. The functional implications and benefits, if any, of this rRNA heterogeneity on translation activity in these diverse cellular contexts, and the associated functional adaptation that may have additionally ensued since the establishment of this mutation in this large group of related archaea is, however, difficult to predict. Replacing the endogenous *E. coli* rRNA population with an rRNA population mimicking the founding mutational event that may have originally occurred in a similarly naïve organism, did not show any significant growth behavior defect (data no shown) (92), and is, in our opinion, unlikely to have/had a major impact on ribosome function. This observation suggests that neutral or nearly neutral rRNA sequence/structure variations may have contributed to a so far underestimated source for the diversification of the ribosome assembly pathway across the tree of life (2,4,6,134). Interestingly, the initial modification observed in archaeal type III organisms may have been randomly sampled across its early founding member population and fixed in most of, if not all, the descendent lineages, thereby suggesting the implementation of yet to be determined beneficial properties during the natural evolution history of these organisms. According to our current knowledge on phylogenetic relationship based on sequences analysis, the presence of a type III G•U wobble in Altiarchaeota is somewhat surprising (110–112). In fact, this common trait suggests that either it may have been acquired independently, or that Altiarchaeota may have shared a common ancestor with Archaeoglobi, Methanogens Class II and Halobacteria. However, the Thermococcalles which would be likely having the same ancestor must have reverted or counter select for this initial change (110–112). Another peculiar aspect is the presence of type III h45 in the recently described Methanonatroarchaea and its absence in Nanohaloarchaeota. Noteworthy, the relative positioning of these organisms is disputed (112,135–137). Whereas our observations are still limited in their scope and depth to validate or invalidate phylogenetic relationship directly, our improved knowledge of ribosome biogenesis diversity in archaea will additionally contribute to refining evolutionary scenarios and phylogenetic relationship, in the form of ‘functional phylogenetic’.

Finally, our 2D RNA prediction analysis of h45 across bacteria and eukaryotes, indicates the presence of additional structural sub-types (Supplementary Figure S4). The putative consequences of this structural diversity, on KsgA/Dim1-dependent methylation and SSU function, is yet to be further explored. Collectively, the analysis presented in this manuscript further supports that molecular diversity may provide a better understanding of how functional adaptability has evolved.

### Substrate conformation-dependent modifications: implication for the KsgA/Dim1 release mechanism

Initial studies in bacteria suggested that KsgA/Dim1′s release from the nascent/matured SSU is closely linked to its catalytic activity and likely occurs after completion of the rRNA methylation reactions (16,31,125). In addition, a recent study in yeast, suggests that Fap7, an adenylate kinase/ATPase, is required for KsgA/Dim1 release (33). Whereas studies in bacteria support a *self-determined* release process, the latter study in Eukaryotes, proposed a factor-dependent assisted release of KsgA/Dim1, thereby, providing an additional layer of regulation during eukaryotic SSU synthesis (16,31,33,125). Interestingly, and in contrast to bacteria, Fap7 sequence homologs can be traced in many archaea including h45 type III-containing organisms [(5) our own observations]. In this study, we provide evidence that methylation completion is not fully required for the KsgA/Dim1 release reactions, suggesting that the modifications-induced h45 structural destabilization and the accompanying SSU structural reorganization correlates with partial/or complete loss of KsgA/Dim1 affinity to the nascent SSU. To determine whether archaeal Fap7 sequence homologs participate in factor-dependent KsgA/Dim1 release *in vivo* in archaea will require additional functional analysis. Even though our current *in vitro* release assay does not allow us yet to fully distinguished between these possibilities, an archaeal Fap7-dependent KsgA/Dim1 release in h45 type-III organism would imply that the Fap7-mediated KsgA/Dim1 release does not directly depend on h45 methylation completion, but rather on the underlying cooperative effects induced by short/long range structural rearrangements stimulated by the h45 destabilization. Future work will be necessary to better understand the respective cooperative effects enabling the regulated h45 methylation and release of KsgA/Dim1 from the nascent SSU in this subset of archaeal organisms. In conclusion, our study further implies that additional molecular diversity across the tree of life influencing KsgA/Dim1 biology, but also ribosome biogenesis in general, remain to be fully explored.

## DATA AVAILABILITY

The mass spectrometry proteomics data have been deposited to the ProteomeXchange Consortium (http://www.proteomexchange.org/) via the PRIDE partner repository (79) with the dataset identifier PXD021827 (doi:10.6019/PXD021827). The data of the mass spectrometry analysis used for the volcano plots, (ar)COG and start codon analyses are provided in Supplementary Tables S4–S6. Scripts used for bioinformatic analysis are available upon request to the corresponding author (sebastien.ferreira-cerca@ur.de).

## Supplementary Material

gkaa1268_Supplemental_Files

## Notes

Present address: Alexander Wagner, Biozentrum, University of Basel, Basel, Switzerland.
