# Supplementary material for: Insights into synthesis and function of KsgA/Dim1-dependent rRNA modifications in archaea
Source: Nucleic Acids Res. 2021 Jan 12;49(3):1662–87. doi: 10.1093/nar/gkaa1268 (PMC7897474; doi:10.1093/nar/gkaa1268)
Supplement: gkaa1268_Supplemental_Files [file gkaa1268_supplemental_files.zip › Supp_figures_rev.pdf]

Insights into synthesis and function of KsgA/Dim1-dependent rRNA modifications in archaea

Robert Knüppel, Christian Trahan, Michael Kern, Alexander Wagner, Felix Grünberger, Winfried Hausner, Tessa E.F. Quax, Sonja-Verena Albers, Marlene Oeffinger and Sébastien Ferreira-Cerca

SUPPLEMENTARY FIGURES 1-4

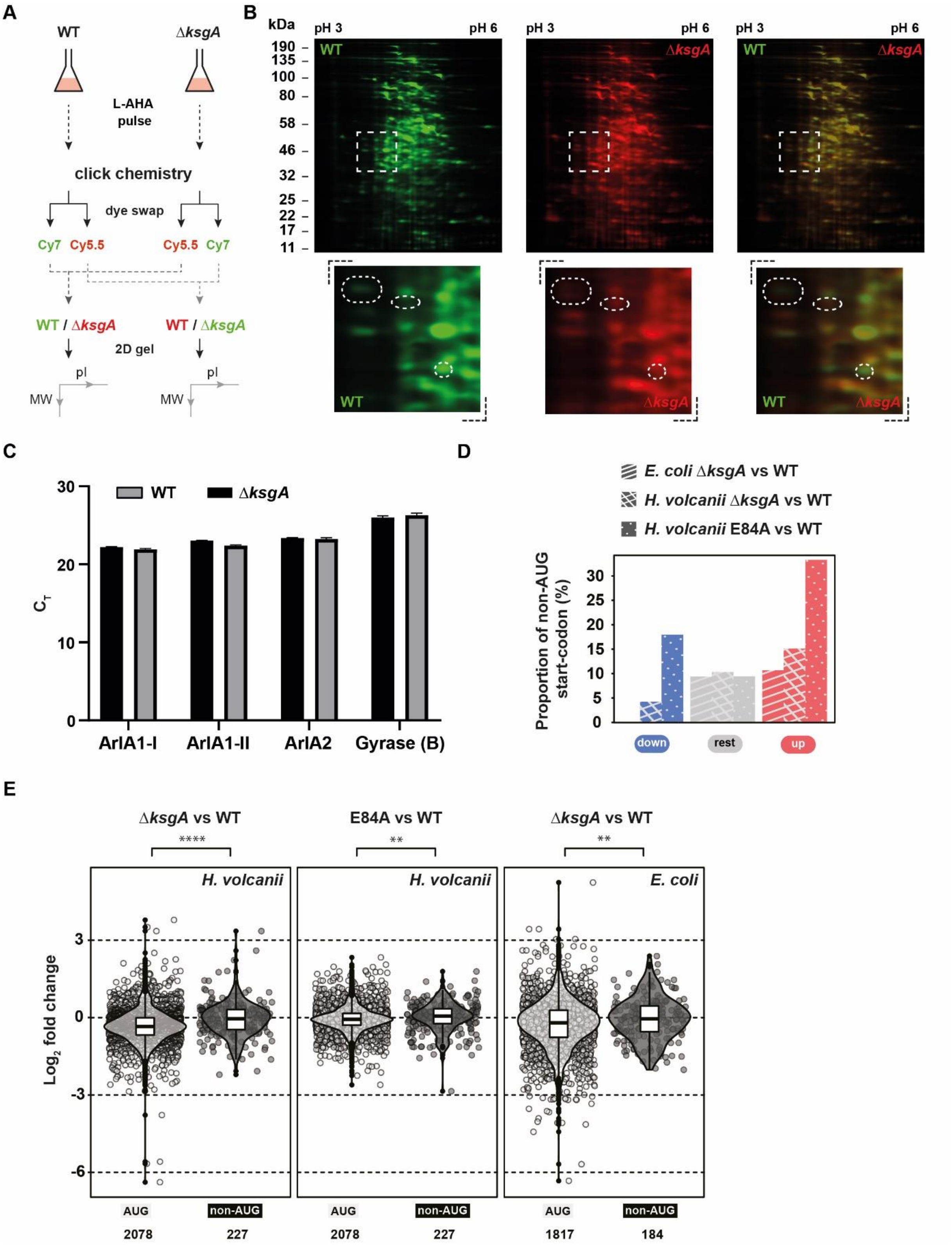

A
WT
ΔksgA
L-AHA pulse
click chemistry
dye swap
Cy7 Cy5.5
Cy5.5 Cy7
WT / ΔksgA
WT / ΔksgA
2D gel
pI
MW
B
kDa
pH 3
pH 6
190
135
100
80
58
46
32
25
22
17
11
WT
ΔksgA
C
WT
ΔksgA
CT
ArlA1-I
ArlA1-II
ArlA2
Gyrase (B)
D
E. coli ΔksgA vs WT
H. volcanii ΔksgA vs WT
H. volcanii E84A vs WT
Proportion of non-AUG start-codon (%)
down
rest
up
E
ΔksgA vs WT
E84A vs WT
ΔksgA vs WT
****
**
**
H. volcanii
H. volcanii
E. coli
Log2 fold change
AUG
non-AUG
2078
227
2078
227
1817
184

Supplementary Figure 1: Effect of KsgA/Dim1 deletion on homeostasis of a subset of proteins.

A) Experimental workflow of 2D BONCAT. Exponentially growing wildtype and KsgA/Dim1 deletion strains were pulse-labelled with 1 mM L-AHA for 45 minutes (representing approx. 20% of the *H. volcanii* doubling time in these growth conditions). Cells were lysed, protein were extracted and subjected to click-chemistry with DBCO-Cy7 and DBCO-Cy5.5. Equal protein amounts of WT cells labelled with DBCO-Cy7 and KsgA/Dim1 cells labelled with DBCO-Cy5.5 and vice-versa (dye-swap to control fluorescence bias) were mixed prior to 2D-SDS-PAGE analysis. Sample mixtures were used to rehydrate immobilized pH stripes. After isoelectric focusing, the second dimension was resolved using 4-12% polyacrylamide gradient gel and the fluorescence signals were acquired with the Li-COR Odyssey Infrared Imager.

B) Exemplary results 2D-BONCAT analysis. Full gel scan for the 2D gel analysis obtained for the wildtype (Cy7-red channel) and mutant *H. volcanii* cells (Cy5.5-green channel) mixture are provided (upper panel). Zoom in of the 2D gel (boxing) showing selected representative differential expression between wildtype and Δ*ksgA/dim1* strains is provided in the lower panel. Please note that dye-swap experiment (WT-Cy5.5/ Δ*ksgA/dim1*-Cy7) showed comparable results (data not shown).

C) Analysis of selected mRNA levels by quantitative RT-PCR. Relative RNA levels for mRNA encoding archaellins (ArlA1, ArlA2), and DNA Gyrase B subunit (HVO_1572) in wildtype and Δ*ksgA/dim1* cells were analyzed by quantitative RT-PCR. Average reported C_T values are plotted. Two different primer pairs (ArlA1-I and ArlA1-II) were used for the analysis of the mRNA level encoding for the major archaealin precursor ArlA1. Note that the reported C_T value differences between wildtype and Δ*ksgA/dim1* cells for the mRNA analyses are <1.

D) Proportion of non-AUG start-codon in differentially expressed proteins.
Proportion (in %) of non-AUG start codon in down-regulated (blue), up-regulated (red), and non-significantly affected (grey, cut-off *p*-value $p < .05$) in the indicated conditions are provided.

E) Start codon usage distribution. Violin plots distribution of start-codon usage (AUG/non AUG) in dependency of differential protein levels in *H. volcanii* WT vs Δ*ksgA/dim1* (left panel) reintegrated WT vs reintegrated *ksgA* E84A (middle panel) and *E. coli* WT vs Δ*ksgA/dim1* (right panel) of the respective identified proteins are provided. The numbers of proteins are indicated. ** $p < .01$ and **** $p < .0001$.

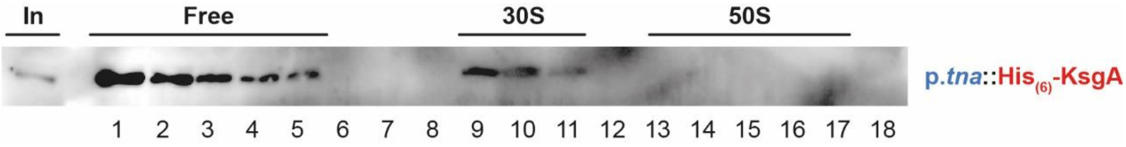

Supplementary Figure 2: *Haloferax volcanii* KsgA/Dim1 co-sediments with the small ribosomal subunit. WCE from Δ*ksgA/dim1* cells complemented with a plasmid carrying KsgA/Dim1 [p.*tna*::$His_{(6)}$-KsgA/Dim1] grown in presence of 0.5 mM tryptophane were separated on sucrose gradient by ultracentrifugation. The gradient was fractionated, and the individual fractions were analyzed by immunoblot using a probe directed against the N-terminal hexa-histidine-tag fused to KsgA/Dim1. Fractions are annotated according to the recorded profile at A_{260nm}.

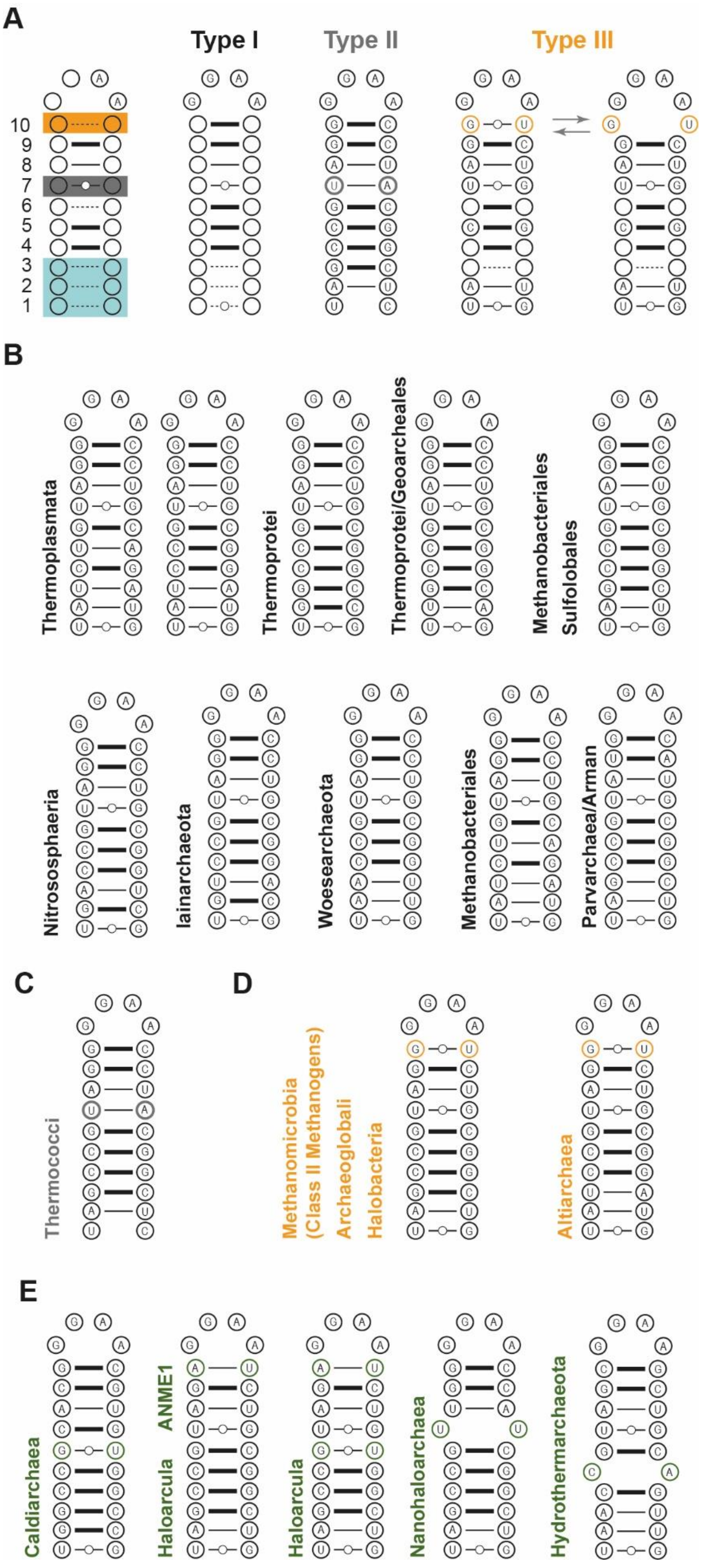
A
Type I
Type II
Type III
10
9
8
7
6
5
4
3
2
1
B
Thermoplasmata
Thermoprotei
Thermoprotei/Geoarcheales
Methanobacteriales
Sulfolobales
Nitrososphaeria
Iainarchaeota
Woesearchaeota
Methanobacteriales
Parvarchaea/Arman
C
Thermococci
D
Methanomicrobia
(Class II Methanogens)
Archaeoglobali
Halobacteria
Altiarchaea
E
Caldiarchaea
Haloarcula
ANME1
Haloarcula
Nanohaloarchaea
Hydrothermarchaeota

Supplementary Figure 3: Exemplary h45 sequence and structure diversity across archaea.

A) 16S rRNA helix 45 secondary structure prediction diversity across archaea. Archaea were classified into 3 major archaeal groups (type I, II and III) based on h45 sequence alignments and structure predictions of representative members of these groups (see **Figure 6**). Base-pairing numbering (1-10) was annotated from the base of the h45 stem. When invariant the corresponding nucleotides and nature of the base pairing (G-C larger line, A-U thinner line or G●U dotted thinner line) are provided. Base pairing variability (G-C, A-U or G●U) is indicated by dashed thinner line. An average representative archaeal h45 structure based on all the compiled sequences (see material and methods) is depicted (upper left). Note that the first 6 nucleotides forming the first 3 base-pairs (indicated in blue) are relatively diverse in comparison to the almost invariant base-pair at position 4-6 and 8/9 across archaea Variability at the G●U base-pairing at position 7 observed in type II and at position 10 specific to the type III group are indicated in grey and orange, respectively.

B) Exemplary 16S rRNA helix 45 secondary structure prediction obtained for archaeal type I groups. Taxonomy level of the respective exemplary sequences and associated secondary structure prediction is provided.

C) Exemplary 16S rRNA helix 45 secondary structure prediction obtained for archaeal type II group. Taxonomy level of the respective exemplary sequences and associated secondary structure prediction is provided.

D) Exemplary 16S rRNA helix 45 secondary structure prediction obtained for archaeal type III groups. Taxonomy level of the respective exemplary sequences and associated secondary structure prediction is provided.

E) Example of unclassified archaeal h45 structural sub-types.

Additional, structural sub-types restricted to a subset of archaeal organisms are provided. Taxonomy level of the respective exemplary sequences and associated secondary structure prediction is provided.

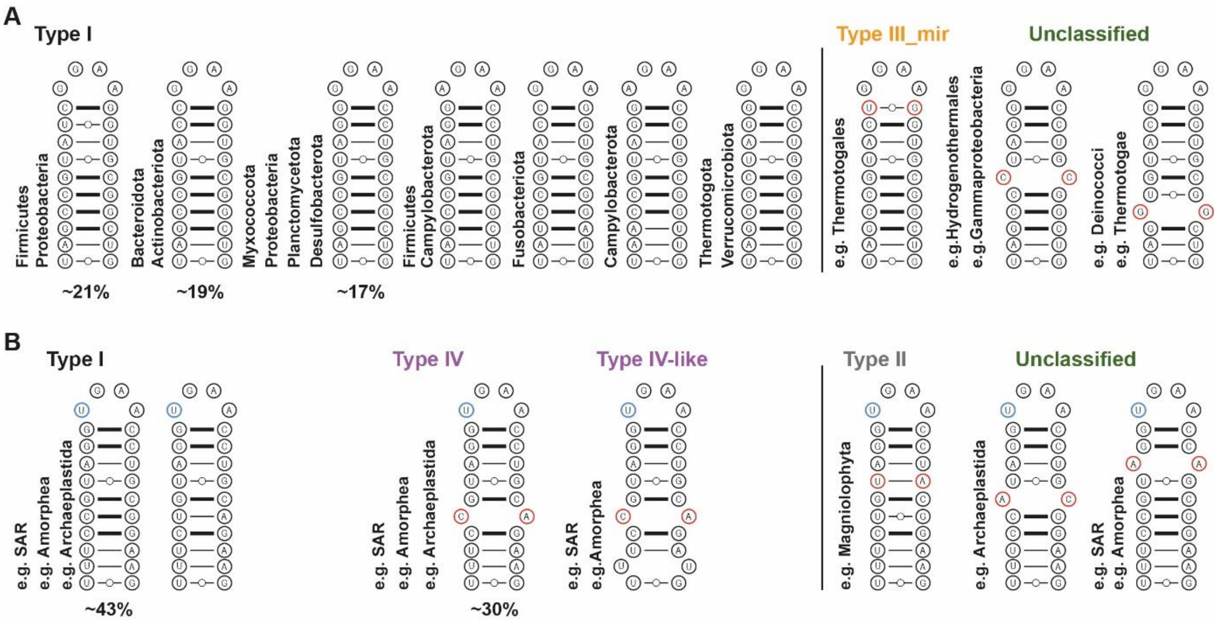

Supplementary Figure 4: A survey of h45 sequence and structure diversity across bacteria and eukaryotes.

Selected exemplary sequences and associated secondary structure prediction predominantly found in bacteria and eukaryotes are provided. Taxonomy level of the respective exemplary sequences and associated secondary structure prediction is indicated. Percentage of the depicted sequence found in the dataset is indicated for the major representative sequences.

A) Exemplary 16S rRNA helix 45 secondary structure prediction obtained for bacterial organisms. Taxonomy level of the respective exemplary sequences and associated secondary structure prediction is provided. Note that most sequences belong to the archetype type I structure. Presence of additional subtype is exemplary illustrated. Note the presence of an h45 structure prediction like the archaeal type III, except that the G●U orientation is mirrored (Type III_mir).

B) Exemplary 18S rRNA helix 45 secondary structure prediction obtained for eukaryotic organisms. Taxonomy level of the respective exemplary sequences and associated secondary structure prediction is provided. Most sequences belong to the archetype type I structure and the additional type IV (non-canonical C●A on position 5 relative to the h45 base). Presence of additional subtypes is illustrated exemplarily. Note the presence of an h45 structure prediction like the archaeal type II (G●U to A-U at position 5 relative to the h45 base). Note that the Eukaryotic h45 tetraloop is characterized by the presence of an almost invariant 5´Uridine absent in archaea and bacteria.
